# Maternal and fetal determinants on kidney size in early childhood: insights from a New York City cohort

**DOI:** 10.1186/s12882-026-04913-1

**Published:** 2026-04-14

**Authors:** Rui Ling, Eunsil Seok, Sarai Encarnacion, Vasuda Kapoor, Mengling Liu, Yelena Afanasyeva, Shailee Lala, Fjolla Hyseni Vokshi, Jie Liu, Laura Malaga-Dieguez, Leonardo Trasande

**Affiliations:** 1 Department of Population Health, New York University Grossman School of Medicine, New York, NY 10010 USA; 2 Department of Pediatrics, New York University Grossman School of Medicine, New York, NY USA; 3 Department of Radiology, New York University Grossman School of Medicine, New York, NY USA; 4 New York University Wagner School of Public Service, New York City, NY USA

**Keywords:** Pediatric nephrology, Brenner hypothesis, Birth weight, Kidney size, Longitudinal study

## Abstract

**Background:**

The role of maternal and fetal characteristics in determining kidney size in early childhood remains largely unexplored. This study aims to evaluate the association between birth weight and kidney size in children aged one to six years and explore other children and maternal determinants in a United States cohort.

**Methods:**

We analyzed data from 892 mother-child pairs enrolled in the New York University Children’s Health and Environment Study (CHES). Renal sonographic measurements were taken from one to six years of age. Kidney size outcomes included average kidney length, width, depth, total kidney volume (TKV), adjusted kidney length (kidney length/body length), and adjusted TKV (TKV/body surface area). Maternal determinants include age, demographic characteristics, pre-pregnancy BMI, lifestyle, pregnancy complications, and diet during pregnancy. Fetal determinants included sex, birth weight for gestational age z-score, and gestational age at delivery. Anthropometric z change and breastfeeding duration were also considered. Associations were examined using crude and covariate-adjusted linear mixed models.

**Results:**

Birth weight z-score and anthropometric z change were observed positively associated with all measures except adjusted kidney length. Female children had smaller average kidney length and TKV, and breastfeeding duration was negatively associated with average kidney depth and TKV. Children of non-Hispanic Black mothers and parous mothers had smaller kidney measures.

**Conclusion:**

In NYU CHES, we found that early childhood kidney size measures were consistently influenced by birth weight z-scores and changes in postnatal weight gain z-scores. Additionally, we observed racial differences and the influence of breastfeeding duration on kidney size.

**Trial registration:**

Not applicable.

**Supplementary Information:**

The online version contains supplementary material available at 10.1186/s12882-026-04913-1.

## Background

Over 35 million adults in the United States were estimated to be affected by chronic kidney disease (CKD) [1]. Having CKD increases the risk of heart disease, stroke, and premature death. The global age-standardized incidence rate of CKD per 100,000 population has increased from 299.06 in 1990 to 310.13 in 2016 [2].

In the 1980s, David Barker introduced the concept that adult diseases might originate in the intrauterine environment, with data demonstrating a link between low birth weight (LBW) and an increased risk of cardiovascular disease [3, 4]. Building on this, Brenner and colleagues proposed that disruptions in renal development due to intrauterine growth restriction (IUGR) and small for gestational age (SGA) status could lead to glomerular hyperfiltration and subsequent hypertension [5]. This hypothesis has been supported by human studies showing an association between LBW and the development of CKD independent of prematurity and smaller kidney size [6–9]. While the 2500 g cutoff for LBW is a useful indicator, birth weight is better understood as a spectrum rather than a dichotomous measure. Bakke and colleagues observed an association between smaller size for gestational age at birth and a smaller kidney volume and lower eGFR at six years old in a perspective population cohort in Netherlands [10].

In addition to birth weight and gestational age, fetal characteristics like sex, fetal head circumference, abdominal circumference, and estimated weight at the third trimester are associated with kidney size in childhood [11, 12]. Postnatal growth also plays an important role in kidney growth. One study with large sample found that weight at 6 months and weight growth postnatally were associated with TKV [10]. More research on postnatal growth is needed. Five studies on breastfeeding and kidney growth showed conflicting results [13–17]. The conflict could be due to the difference of long-term and short-term effect of breastfeeding [16].

For maternal characteristics, most research focuses on nutrition during pregnancy and offspring kidney structure or function, but results have not been consistent [18]. Geelhoed also found that maternal anthropometrics before pregnancy were associated with kidney volume [11]. A case-control study has shown associations between childhood CKD and maternal GDM, overweight, and obesity [19]. There is a lack of research examining comprehensive maternal factors such as pregnancy complications and sociodemographic characteristics on sonographic kidney size, which are known factors associated with fetal growth.

This study aims to explore the association of kidney growth up to six years with fetal determinants, such as birth weight and gestational age, maternal determinants, and postnatal growth in a United States (U.S.) birth cohort.

## Methods

### Setting and participants

The New York University (NYU) Children’s Health and Environment Study (CHES) is a prospective birth cohort in New York City (NYC) with participants from a broad range of backgrounds [20]. To be eligible for enrollment, participants must be at least 18 years old, less than 18 weeks pregnant, have a pregnancy that is not medically threatened, and plan to deliver at one of the three NYU-affiliated hospitals (NYU Langone Hospital—Manhattan, Bellevue Hospital, and NYU Langone Hospital—Brooklyn) [20]. Sociodemographic characteristics, medical history, and pregnancy experiences were collected through initial enrollment questionnaires and follow-up surveys during pregnancy [20]. After the children were born, postpartum visits occurred at the following intervals: 12–23, 24–35, 36–59, and 60–71 months of age, where sonographic measurements were recorded for children. Additionally, maternal and child health history data were obtained directly from electronic medical records (EMR). This analysis was limited to singleton live-born children who had participated in at least one measurement. Children with kidney abnormalities were excluded (*n* = 9). The NYU CHES study was conducted in accordance with the ethical standards of the Declaration of Helsinki and was approved by the Institutional Review Board of the NYU Grossman School of Medicine. All participants in CHES were informed about the study’s objectives, risks, and the voluntary nature of their participation. All maternal participants provided written informed consent at the time of enrollment and additional consent for postpartum follow-up.

### Outcomes: kidney size

Renal ultrasounds were repeatedly performed in children during the postpartum visits using standard devices (Affiniti-70, Phillips) with a curved probe (C9-2, Phillips). The ultrasound examinations were performed in a clinical setting. The majority of children were positioned in supine and lateral decubitus positions. Positional accommodation was provided for some of the children younger than 24 months and children with special needs. Accommodation included image acquisition while children were held in their guardians’/parents’ arms in an upright position and while children were standing up. During the visit, toys and distraction devices were used to help comfort the child.

Sonographers acquired multiple longitudinal and transverse greyscale and color Doppler images of the kidneys. The sonographic images were then assessed by a pediatric radiologist (SL) who measured each kidney in three dimensions: longitudinal diameter (length), anterior-posterior diameter (depth), and transverse diameter (width). Up to three measurements were provided for each dimension. Imaging review with quantitative measurements was performed on *syngo.via* (Siemens-Healthineers, Germany).

The average kidney dimensions of length, depth, and width were calculated by averaging the respective measurements of the left and right kidneys.Left and right kidney volume was estimated using the ellipsoid equation: volume (cm^3) = 0.523 x length (cm) x width (cm) x depth (cm). Total kidney volume (TKV) was derived by summing the volumes of left and right kidneys. To account for postnatal growth, adjusted kidney length and volume were computed. Adjusted TKV was defined as the ratio of TKV to body surface area (ml/m^2). Adjusted kidney length was calculated as the average of adjusted left and right kidney lengths, with each adjusted by dividing the respective kidney length by body length (mm/cm).

To assess inter-reader reliability, a second radiologist (FHV) performed an independent and blinded review of the images from every tenth visit, recording renal length, depth and width. The original readings and QC readings were entered and stored in the REDCap project by a sonographer who was not involved in the reading (SE) and all files were reviewed for completeness and data entry errors. The Intraclass Correlation Coefficients 2 (ICC2) were calculated using a two-way random effects model for each renal measurement. The *irr* package in R was utilized with model = “*twoway*”, type = “*agreement*” and unit = “*single*”. ICC2s for all six measurements were above 0.96 (Table S8).

### Predictors

Fetal determinants included sex, birth weight (g), and gestational age (weeks) at delivery. Birth weight for gestational z-score (birth weight z-score) were calculated using the INTERGROWTH-21st standard which accounts for gestational age and sex [21]. We also considered preterm birth (PTB, < 37 weeks), LBW (≤ 2500 g), and small for gestational age (SGA, birth weight < 10th percentile for their gestational age and sex).

Child weight-for-age (WFA) z-score and breastfeeding during the first year were considered as factors to account for postnatal growth. WFA z-score based on the WHO growth standard was calculated using the *who_wtkg2zscore* function in the *growthstandards* R package [22]. Age correction was conducted for WFA z-score among children who were born preterm through 36 months by subtracting the number of weeks born before 40 weeks of gestation [23]. We also calculated the anthropometric z change since WFA Z-score and birth weight Z-score are highly correlated. with the formula: *anthropometric z change = WFA Z-score - BW Z-score*. For a child with birth weight z-score of 1.35 and WFA z-score of 0.31, the anthropometric z change would be -1. Breastfeeding duration in days was calculated based on self-reported questionnaire data collected at multiple time points (from 4 to 24 months). Durations longer than 365 days were capped at 365 days. Breastfeeding duration was further categorized as less than 6 months or 6 months or more.

Maternal sociodemographic characteristics, psychosocial factors, substance use, diet, and pregnancy complications were considered as maternal determinants. Maternal race and ethnicity (Hispanic, non-Hispanic White, non-Hispanic Black, non-Hispanic Asian, and other), marital status (married/living with a partner, divorced/separated/single/widowed), education (high school or less, some college but no degree/associate degree, bachelor degree, post-graduate degree), employment status, maternal age at enrollment, self-reported smoking during pregnancy, alcohol during pregnancy were collected using CHES questionnaires.

Maternal dietary habits were assessed during pregnancy using the validated Diet History Questionnaire II (DHQ II) [24]. The DHQ II asked about the frequency and portion size of food intake over the past year, which covered part of pregnancy and the pre-pregnancy period. Intake of total energy (kcal), protein (g), retinol (mcg), folate (mcg), sodium(mg), and potassium (mg) were calculated [25]. Due to the skewness of the distribution of the food intake, the food intake was natural-log transformed. The Healthy Eating Index-2015 (HEI) was calculated to quantify alignment with the 2015–2020 Dietary Guidelines for Americans [26].

Parity, insurance status (private and public), maternal age at enrollment, pre-pregnancy body mass index (BMI, kg/m2), pre-eclampsia, eclampsia, gestational hypertension, superimposed preeclampsia, chronic hypertension, and gestational diabetes mellitus (GDM) were collected from EMR with permission from participants. We created a hypertension during pregnancy (HDP) variable as a composite of chronic hypertension, preeclampsia, eclampsia, gestational hypertension, and superimposed preeclampsia. Pre-pregnancy BMI was categorized as underweight and normal (< 25 kg/m^2), overweight (25 to < 30 kg/m^2), and obese (≥ 30 kg/m^2).

### Statistical analysis

Summary statistics were calculated for maternal and child characteristics. Boxplots were used to visualize the distribution of the continuous variables (Figure S1 & S2). The mean and standard deviations (SD) were reported for maternal age and birth weight z-scores. For other continuous variables that were not normally distributed, medians and interquartile ranges (IQR) were presented. Categorical variables were summarized using frequencies and percentages. The Kruskal-Wallis test and Chi-square test were used to compare the analytic sample with those who were eligible but lost to follow-up. Additionally, the mean and SD were reported for child kidney size outcomes and postnatal growth measurements.

Crude associations between each determinant and each of the longitudinal kidney size outcomes were analyzed using linear mixed-effect models (LMM). A unique participant ID was included as a random effect, and child age in months at each visit was included to account for the longitudinal trend. Determinants with a crude p-value of < 0.1 for any of the kidney size outcomes were considered as independent variables in the adjusted models. To assess collinearity, a correlation coefficient matrix was created for all continuous variables (Figure S3). Subsequently, two sets of adjusted models were fitted to explore the association between the determinants and each of the kidney size outcomes. The first set of adjusted LMMs (Model 1) included fetal determinants (child sex, birth weight z-score, and GA) and maternal characteristics that were selected from crude models. The second set of adjusted LMMs (Model 2) added postnatal factors to Model 1. All variables included in the adjusted models had < 4% missing data, and we performed complete case models.

Models were further limited to participants who reported no smoking during pregnancy as only six participants reported smoking during this period. Additionally, we conducted the following sensitivity analyses with fully-adjusted Model 2: (1) using the primary analysis sample but adding a quadratic term for time (child age in months); (2) excluding those with PTB; (3) excluding those with LBW; (4) excluding SGA; (5) using a subset of the analytical sample, we assessed the association between cotinine levels, treated as a continuous variable, and outcome measures. Details of the cotinine levels are included in Table S6. All statistical analyses were performed using R version 4.4.0.

## Results

**Fig. 1:**
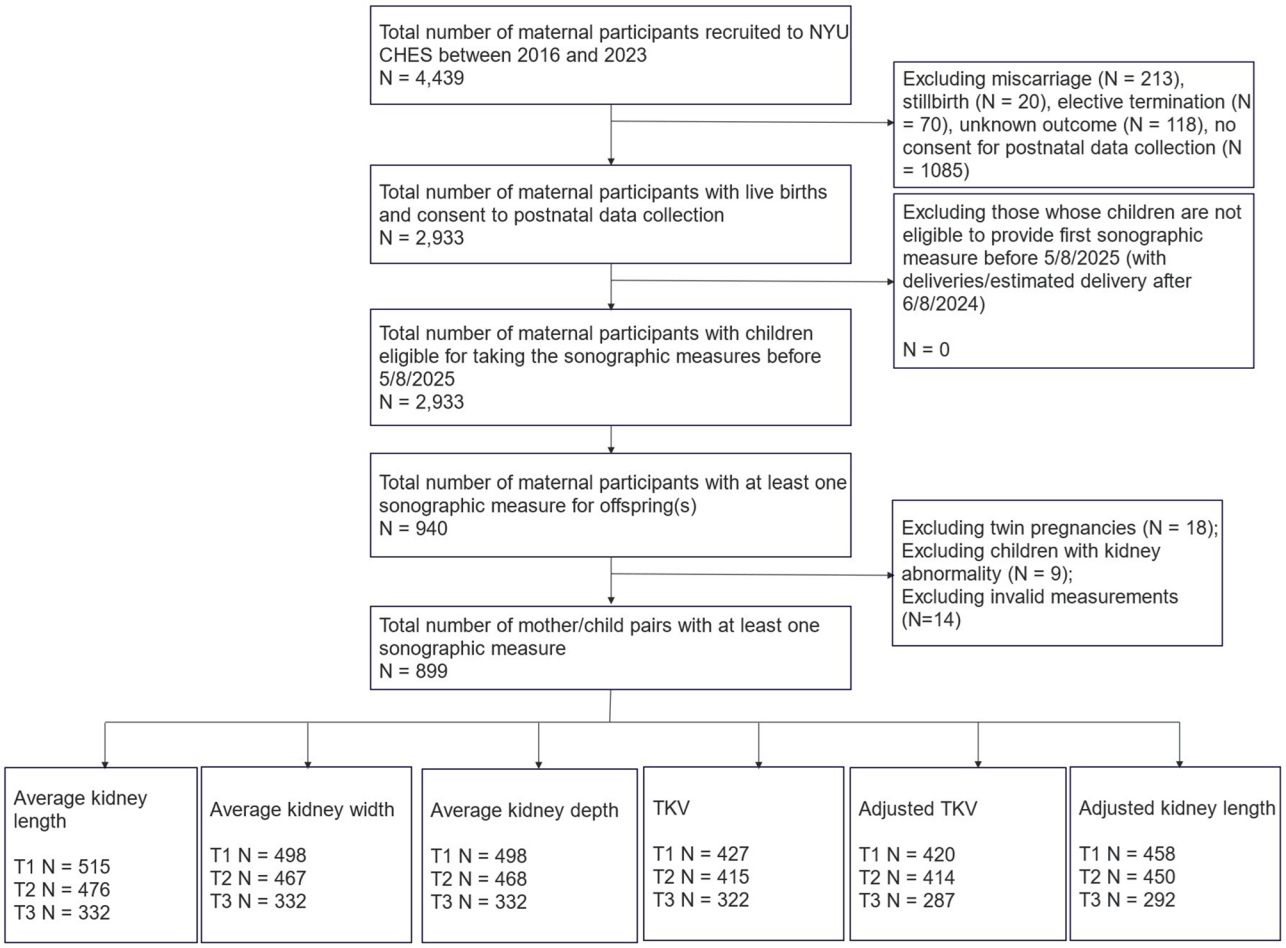
Sample size flowchart. All children of maternal participants enrolled in the New York University Children’s Health and Environment Study from 2016 to 2023 were eligible to start taking sonographic measures at 11 months of age

Among a total of 2933 maternal participants enrolled in CHES between 2016 and 2023 who consented to postnatal follow-up, 940 participants had their children participate in the sonographic scan for kidney size outcomes. After excluding 18 twin pregnancies, nine children with kidney abnormality, and 14 participants with invalid measurements, the final analysis included 899 singleton mother-child pairs (Fig. 1). Among the 899 children, 378 (42%) had more than one visit. A comparison between the analytic sample and those that are eligible (Table S7).

Among the 899 mothers, only six participants (0.7%) reported smoking during pregnancy and one missing smoking status. Due to this small percentage for smoking, the primary analysis was restricted to the 892 pairs with no smoking during pregnancy. Among the final analytic sample (*N* = 892), 66% self-identified as Hispanic, 21.3% identified as non-Hispanic White, 7.2% as non-Hispanic Asian, and 3.6% as non-Hispanic black (Table 1). The mean age at enrollment was 31.8 years (SD: 5.6). Approximately 40% of the mothers had pre-pregnancy BMI categorized as underweight or normal weight. Most mothers were married or living with a partner (87.7%). About half of the mothers received a high school degree or less (47.1%) and were employed (54.1%). About 65% of mothers had public insurance, and around 37% were first-time mothers (nulliparous). Regarding pregnancy complications, 26.6% had GDM and 16.7% were diagnosed with at least one type of HDP. Nearly 10% screened positive for anxiety and depression during pregnancy.

**Table 1:** Participants characteristics (*N*=892)

	*N*(%) *
**Fetal characteristics**	
Child sex: Female	430 (48.2%)
Birth weight (g): Median (IQR)	3313.5 (610)
Birth weight z-score *: Mean (SD)	0.27 (0.96)
Gestational age at birth (week): Median (IQR)	39.29 (1.79)
PTB	66 (7.4%)
LBW	62 (7.0%)
SGA	46 (5.2%)
**Maternal characteristics**	
Maternal age (year): Mean (SD)	31.77 (5.65)
Pre-pregnancy BMI (kg/m^2): Median (IQR)	26.42 (7.5)
Underweight/Normal weight	348 (40.2%)
Overweight	277 (32.0%)
Obese	241 (27.8%)
Maternal race/ethnicity	
Hispanic	582 (65.9%)
Non-Hispanic White	188 (21.3%)
Non-Hispanic Black	32 (3.6%)
Asian	64 (7.2%)
Other/Multiple race	17 (1.9%)
Marital status	
Married/living with a partner	781 (87.7%)
Divorced/separated/single/widowed	110 (12.3%)
Education	
High school or less	413 (47.1%)
Some college but no degree/Associate degree	138 (15.7%)
Bachelor’s degree	160 (18.2%)
Post-graduate degree	166 (18.9%)
Employed	483 (54.1%)
Insurance type	
Public	568 (65.0%)
Private	306 (35.0%)
Parity	
Nulliparous	332 (37.3%)
Parous	558 (62.7%)
GDM	237 (26.6%)
HDP	149 (16.7%)
Chronic hypertension	36 (4.0%)
Preeclampsia	85 (9.6%)
PIH	49 (5.5%)
Depression	88 (9.9%)
Anxiety	73 (8.2%)
Alcohol use during pregnancy	146 (16.4%)
**Breastfeeding duration**	
Less than 6 months	335 (38.8%)
More than 6 months	529 (61.2%)
**Maternal Diet during pregnancy**	
Energy (kcal): Median (IQR)	1432.64 (1088.53)
Protein (g): Median (IQR)	53.64 (48.77)
Retinol (mcg): Median (IQR)	292.88 (305)
Folate (mcg): Median (IQR)	375.28 (309.54)
Sodium (mg): Median (IQR)	2137.29 (1858.43)
Potassium (mg): Median (IQR)	2552.92 (2052.17)
HEI total score: Median (IQR)	72.37 (10.93)

Notes: For maternal age and birth weight Z-score, mean (SD) is provided. For other continuous variables, median (IQR) is provided. For categorical variables, number (percentage) is provided. Birth weight z-scores are sex and gestational week dependent and were calculated through INTERGROWTH-21st tool

Missing data, n(%): Birth weight (g), 12 (1.35%); Birth weight z-score, 13 (1.46%); LBW, 12 (1.35%); SGA, 13 (1.46%); Pre-pregnancy BMI (kg/m^2), 26 (2.91%); Maternal race/ethnicity, 9 (1.01%); Marital status, 1 (0.11%); Education, 1 (1.68%); Insurance type, 18 (2.02%); Parity, 2 (0.22%); GDM, 2 (0.22%); HDP, 2 (0.22%); Chronic hypertension, 2 (0.22%); Preeclampsia, 2 (0.22%); PIH, 2 (0.22%); Depression, 2 (0.22%); Anxiety, 2 (0.22%); Alcohol use during pregnancy, 2 (0.22%); Maternal diet during pregnancy, 141 (15.81%); Breastfeeding duration, 28 (3.14%)

Among the 892 children, about half of them were girls (48.2%). The mean birth weight z-scores was 0.27 (SD: 0.96), and the median GA was 39.29 weeks (IQR: 1.79). Approximately 39% of the children had < 6 months breastfeeding duration.

For kidney outcomes at the first timepoint, the average age was 15 months with a SD of 3.8 months. The mean (SD) of average kidney length, width, and depth were 6.2 (0.56) cm, 3.07 (0.4) cm, and 3.22 (0.36) cm, respectively. The mean (SD) TKV was 65.16 (16.50) ml. After adjusting for body size, the mean (SD) adjusted TKV was 140.97 (27.7), and the mean (SD) adjusted kidney length was 0.80 (0.06) mm/cm. The average age of the second timepoint was 27 months (SD: 3.67 months) and the average of the third timepoint was 54 months (SD 15.6 months). The mean (SD) values for kidney length, width, depth, TKV, and adjusted TKV increased over time, while the adjusted kidney length decreased over time (Table 2, Figure S2).

**Table 2:** Summary statistics of child kidney size outcome and postnatal growth per timepoint: Mean (SD)

	Male	Female	Total
**Timepoint 1 (N)**	257	261	518
Age at timepoint (month)	14.98 (3.83)	14.95 (3.79)	14.96 (3.81)
Weight for age z-score	0.47 (1.04)	0.54 (1.00)	0.51 (1.02)
Average kidney length (cm)	6.23 (0.60)	6.13 (0.51)	6.18 (0.56)
Average kidney width (cm)	3.09 (0.45)	3.05 (0.34)	3.07 (0.40)
Average kidney depth (cm)	3.24 (0.36)	3.20 (0.36)	3.22 (0.36)
TKV (mL)	66.04 (17.45)	64.30 (15.48)	65.16 (16.50)
TKV/BSA (mL/m^2)	139.63 (28.25)	142.29 (27.14)	140.97 (27.70)
KBR: average (mm/cm)	0.80 (0.06)	0.80 (0.06)	0.80 (0.06)
**Timepoint 2 (N)**	250	226	476
Age at timepoint (month)	27.38 (3.69)	27.27 (3.65)	27.33 (3.67)
Weight for age z-score	0.57 (1.18)	0.69 (1.17)	0.63 (1.18)
Average kidney length (cm)	6.76 (0.55)	6.70 (0.70)	6.73 (0.63)
Average kidney width (cm)	3.30 (0.34)	3.33 (0.54)	3.31 (0.45)
Average kidney depth (cm)	3.47 (0.36)	3.47 (0.34)	3.47 (0.35)
TKV (mL)	82.76 (20.04)	81.66 (18.90)	82.24 (19.49)
TKV/BSA (mL/m^2)	144.33 (27.33)	145.46 (26.73)	144.86 (27.02)
KBR: average (mm/cm)	0.75 (0.05)	0.76 (0.06)	0.76 (0.06)
**Timepoint 3 (N)**	183	149	332
Age at timepoint (month)	53.44 (15.16)	54.54 (16.15)	53.93 (15.60)
Weight for age z-score*	0.91 (1.56)	0.54 (1.26)	0.74 (1.44)
Average kidney length (cm)*	7.57 (0.65)	7.44 (0.65)	7.51 (0.65)
Average kidney width (cm)*	3.64 (0.37)	3.56 (0.40)	3.61 (0.39)
Average kidney depth (cm)*	3.89 (0.37)	3.82 (0.39)	3.86 (0.38)
TKV (mL)*	115.01 (28.84)	107.07 (29.14)	111.47 (29.20)
TKV/BSA (mL/m^2)*	153.43 (27.08)	145.72 (24.56)	150.05 (26.25)
KBR: average (mm/cm)	0.72 (0.05)	0.72 (0.05)	0.72 (0.05)

Note: * indicates significant difference between male and female. We combined two postnatal visits to Timepoint 3. Participants might have more than one measurement in Timepoint 3. The data presented in the table is the median of all at each timepoint

Missing data for three timepoint in order, n(%): Weight for age z-score, 4 (0.77%), 2 (0.41%), 33 (9.94%); Average kidney length (cm), 3 (0.58%), 0, 0; Average kidney width (cm), 20 (3.86%), 9 (1.89%), 0; Average kidney depth (cm), 20 (3.86%), 8 (1.68%), 0; TKV (mL), 91 (17.57%), 61 (12.82%), 10 (3%); TKV/BSA (mL/m^2), 98 (18.92%), 62 (13.03%), 45 (13.55%); KBR: average (mm/cm), 60 (11.58%), 26 (5.46%), 40 (12%)

### Maternal and fetal determinants

Maternal characteristics such as age, pre-pregnancy BMI, maternal race & ethnicity, education, and parity were associated with one or more kidney size outcomes with a p-value less than 0.05 in the single-determinant models (Table S1). All fetal characteristics, including child sex, birth weight z-score, and GA were also associated with one or more kidney outcomes in the single-determinant models (Table S1).

In Model 1 (Table 3), all maternal and fetal characteristics with a p-value less than 0.05 were included. birth weight z-score remained associated with all kidney size outcomes except for adjusted kidney length. For one SD increase in birth weight z-score, average kidney length increased by 0.1 cm (95% CI: 0.06, 0.14), average kidney width increased by 0.05 cm (95% CI: 0.02, 0.08), average kidney depth increased by 0.07 cm (95% CI: 0.04, 0.09), TKV increased by 4.55 ml (95% CI: 3.15, 5.96), and adjusted TKV increased by 2.54 ml/m^2 (95% CI: 0.53, 4.54). Female offspring had smaller kidneys with shorter average kidney length (-0.11; 95% CI: -0.18, -0.03) and lower TKV (-3.15; 95% CI: -5.7, -0.60) after adjusting for other covariates.

**Table 3:** Model 1 - Adjusted LMMs for kidney outcomes (adjusted for maternal and fetal determinants)

	Average Kidney Length (*N*=836)		Average Kidney Width (*N*=823)		Average kidney depth (*N*=824)		TKV (*N*=767)		Adjusted TKV (*N*=739)		Adjusted Kidney Length (*N*=774)	
	Estimate	95% CI	Estimate	95% CI	Estimate	95% CI	Estimate	95% CI	Estimate	95% CI	Estimate	95% CI
Intercept	**5.333**	**(4.39**,** 6.279)**	**2.685**	**(2.033**,** 3.337)**	**3.059**	**(2.51**,** 3.61)**	**39.985**	**(7.368**,** 72.711)**	**134.756**	**(88.491**,** 181.244)**	**0.789**	**(0.695**,** 0.882)**
Child age at visit (month)	**0.031**	**(0.03**,** 0.033)**	**0.013**	**(0.012**,** 0.014)**	**0.016**	**(0.015**,** 0.017)**	**1.143**	**(1.088**,** 1.198)**	**0.351**	**(0.252**,** 0.448)**	**-0.002**	**(-0.002**,** -0.002)**
Child sex: female	**-0.108**	**(-0.182**,** -0.033)**	-0.031	(-0.082, 0.021)	-0.023	(-0.066, 0.02)	**-3.147**	**(-5.703**,** -0.598)**	-0.393	(-4.015, 3.22)	0.005	(-0.003, 0.012)
Birth weight z-score *	**0.104**	**(0.063**,** 0.144)**	**0.051**	**(0.023**,** 0.079)**	**0.065**	**(0.042**,** 0.089)**	**4.553**	**(3.15**,** 5.956)**	**2.538**	**(0.533**,** 4.542)**	0	(-0.004, 0.004)
Gestational age	0.011	(-0.012, 0.034)	0.011	(-0.005, 0.027)	0	(-0.014, 0.013)	0.413	(-0.384, 1.206)	0.264	(-0.87, 1.394)	0.001	(-0.002, 0.003)
Maternal age	0.001	(-0.006, 0.008)	-0.004	(-0.009, 0.001)	-0.001	(-0.005, 0.003)	-0.156	(-0.406, 0.094)	-0.28	(-0.636, 0.077)	0	(-0.001, 0.001)
Pre-pregnancy BMI												
Overweight	0.013	(-0.08, 0.105)	0	(-0.064, 0.064)	**0.057**	**(0.003**,** 0.111)**	2.463	(-0.719, 5.645)	3.618	(-0.894, 8.133)	-0.002	(-0.011, 0.008)
Obese	0.036	(-0.064, 0.136)	0.01	(-0.059, 0.079)	0.047	(-0.011, 0.104)	2.265	(-1.156, 5.674)	0.675	(-4.152, 5.494)	0.002	(-0.008, 0.012)
Race/ethnicity												
Non-Hispanic White	-0.099	(-0.236, 0.038)	-0.034	(-0.128, 0.06)	-0.039	(-0.118, 0.041)	-2.693	(-7.389, 2.004)	-1.122	(-7.749, 5.495)	-0.005	(-0.018, 0.008)
Non-Hispanic Black	-0.123	(-0.339, 0.093)	0.077	(-0.074, 0.228)	0.024	(-0.104, 0.152)	1.139	(-6.254, 8.546)	0.066	(-10.967, 11.136)	**-0.028**	**(-0.05**,** -0.005)**
Asian	-0.073	(-0.249, 0.104)	-0.067	(-0.19, 0.055)	-0.073	(-0.176, 0.03)	-4.487	(-10.595, 1.62)	-3.145	(-11.872, 5.564)	-0.012	(-0.029, 0.006)
Other/Multiple race	-0.162	(-0.451, 0.127)	-0.074	(-0.279, 0.131)	-0.151	(-0.325, 0.022)	-5.272	(-15.509, 4.977)	-5.39	(-20.683, 9.909)	-0.014	(-0.044, 0.015)
Education												
Some college/Associate degree	-0.025	(-0.137, 0.087)	-0.02	(-0.097, 0.057)	-0.034	(-0.098, 0.031)	-2.012	(-5.821, 1.802)	-3.334	(-8.72, 2.059)	-0.007	(-0.018, 0.004)
Bachelor’s degree	0.035	(-0.103, 0.173)	-0.048	(-0.143, 0.047)	-0.005	(-0.085, 0.076)	-1.345	(-6.091, 3.392)	-1.922	(-8.699, 4.854)	0.003	(-0.01, 0.017)
Post-graduate degree	0.054	(-0.101, 0.208)	-0.044	(-0.15, 0.063)	0.015	(-0.075, 0.105)	-0.968	(-6.29, 4.346)	-1.201	(-8.723, 6.317)	-0.001	(-0.016, 0.014)
Parity: Parous	0.042	(-0.047, 0.132)	**-0.083**	**(-0.144**,** -0.021)**	**-0.055**	**(-0.107**,** -0.003)**	-2.219	(-5.312, 0.869)	-3.167	(-7.543, 1.202)	0.004	(-0.005, 0.012)

Notes: All models include time (age at visit in month) to adjust for child age; Bolded effect indicates statistical significance with a=0.05. TKV is the total kidney volume for right and left kidney. Length, width, depth are average of left and right kidney measurement. Adjusted TKV is the ratio of TKV and body surface area. Adjusted kidney length is the ratio of kidney length and child height. Birth weight z-scores are sex and gestational week dependent and were calculated through INTERGROWTH-21st tool

For maternal characteristics, children of overweight mothers had larger kidneys with a 0.06 cm increase in average kidney depth (95% CI: 0.003, 0.11) in comparison to mothers with normal weight or underweight. Children of non-Hispanic black mothers had smaller adjusted kidney length (-0.03; 95% CI: -0.05, -0.01) compared to children of Hispanic mothers. Compared with nulliparous participants, parous women had children with smaller average kidney width (-0.08; 95% CI: -0.14, -0.02) and smaller average kidney depth (-0.06, 95% CI: -0.11, -0.003).

### Postnatal growth

In the single-determinant analysis, breastfeeding duration, WFA z-score, and anthropometric z change were all associated with four or five of the six kidney outcomes.

In Model 2 (Table 4), we added breastfeeding and anthropometric z change to the model. The effect of child sex on average kidney length (-0.1; 95% CI: -0.18, -0.03) and TKV (-2.6; 95% CI: -4.77, -0.44) remained statistically significant but were closer to null compared to Model 1. birth weight z-score remained significantly associated with average kidney length (0.2; 95% CI: 0.16, 0.24), average kidney width (0.12; 95% CI: 0.09, 0.15), average kidney depth (0.13; 95% CI: 0.11, 0.16), TKV (9.12; 95% CI: 7.79, 10.47), and adjusted TKV (4.39; 95% CI: 2.19, 6.61). Children who were breastfed longer than six months tend to have smaller average kidney depth (-0.05; 95% CI: -0.09, -0.004) and adjusted TKV (-4.89; 95% CI: -8.63, -1.15). Anthropometric z change was positively associated with all kidney size outcomes except for adjusted kidney length. For one SD increase in anthropometric z change, average kidney length increased by 0.17 cm (95% CI: 0.14, 0.2), average kidney width increased by 0.13 cm (95% CI: 0.11, 0.15), average kidney depth increased by 0.12 cm (95% CI: 0.1, 0.13), TKV increased by 8.27 ml (95% CI: 7.33, 9.23), and adjusted TKV increased by 2.88 ml/m^2 (95% CI: 1.34, 4.48).

For maternal characteristics, children of non-Hispanic black mothers continued to have significantly smaller adjusted kidney length compared to children of Hispanic mothers (-0.03; 95% CI: -0.05, -0.01).

### Sensitivity analyses

Results from the sensitivity analyses (1) to (3) were consistent with the primary results for all determinants (Table S2 to S4). For sensitivity analyses (4), we observed overall consistent with the primary analysis, and additionally (Table S5), we observed that mothers self-identified as other or multiple races had children with smaller kidney depth (-0.18, 95% CI: -0.36, -0.002).

Another sensitive analysis was conducted to further explore the effect of smoking (Table S6). Model 2 was run with an additional variable of log2-transformed urine cotinine levels with a sub-sample that have urine cotinine levels available during pregnancy. We observed consistent results with birth weight Z-score, maternal race/ethnicity, parity, and anthropometric z change. A negative association was found between cotinine levels (log2-transformed) and average kidney length (-0.024; 95% CI: − 0.05, − 0.002).

## Discussion

In this study, we observed that birth weight Z-score and anthropometric z change were positively associated with kidney size across five kidney size outcomes after adjusting for GA, child sex, breastfeeding duration, and maternal determinants including pre-pregnancy BMI, race and ethnicity, and parity. Our findings support our hypotheses that birth weight is associated with kidney size even among a healthy birth cohort, extending the Brenner’s hypothesis to children with normal birth weight; [5] and postnatal growth is a factor contributing to the association between birth weight and kidney size. Additionally, maternal race and ethnicity, parity, and breastfeeding duration during the first year were observed to be associated with child kidney size.

Little is known about postnatal growth and kidney development. One study in The Netherlands found an association between weight at six months and TKV in childhood [10]. In our study, both WFA z-score and postnatal weight gain measured by the anthropometric z change were assessed using the weight at the age of the kidney measure. Both WFA z-score and anthropometric z change were shown to be significantly associated with kidney size in early childhood. While this measure of anthropometric z change is not ideal for interpretation, it represents net growth after birth. In model 2, even when adjusting for birth weight z-score and other covariates, anthropometric z change still contributed to the difference in kidney size. Future research in kidney development should take the effect of child growth into account.

Previous studies outside the U.S. have found racial and ethnic differences in kidney size [11, 19, 27], and breastfeeding is associated with kidney size positively [13–17]. This study is of the first studies examining maternal predictors of kidney size outcomes in the U.S. Our findings suggested that there are racial differences in offspring kidney size. Children of mothers who are Black might have slightly smaller kidneys. In our analysis, race and ethnicity were included as determinants that might be associated with kidney morphology, with an emphasis on their roles as social constructs that are closely linked to exposure to racism, differential access to healthcare, neighborhood environment, and other socioeconomic determinants of health, rather than to genetic or biological differences [28, 29]. For breastfeeding, we observed an inverse association between breastfeeding more than 6 months in the first year and kidney depth and adjusted TKV. This finding aligned with two studies comparing the effect of formula-fed and breastfed infants on kidney growth that suggests formula-fed children have bigger kidney and better kidney function in early childhood [15, 17].

Height-adjusted kidney length has been historically used among pediatric nephrologists as a standard method to evaluate kidney size in children. Here we explored multiple outcome measures beside adjusted kidney length (kidney length/body length). Our finding suggests that adjusted kidney length might be less sensitive compared to the other kidney outcomes. In our findings, various factors are more consistently associated with kidney dimensions, TKV, and adjusted TKV, but not with adjusted kidney length.

Notable strengths of this study include the availability of prospective repeated sonographic measures over six years in early childhood, which allows for a longitudinal examination of associations. To our knowledge, this is one of the first cohort studies in the U.S. to explore determinants predicting kidney size in early childhood using prospective repeated measures that encompass a wide range of child and maternal determinants. Regarding data quality, all ultrasounds were conducted by licensed sonographers in a clinical setting with a rigorous quality control process, and most determinants were derived from EMR or prospective assessments.

**Table 4:** Model 2 - Adjusted LMMs for kidney outcomes (adjusted for maternal, fetal determinants, breastfeeding duration, and Anthropometric z change *)

	Average Kidney Length (*N*=787)		Average Kidney Width (*N*=774)		Average kidney depth (*N*=775)		TKV (*N*=721)		Adjusted TKV (*N*=716)		Adjusted Kidney Length (*N*=746)	
	Estimate	95% CI	Estimate	95% CI	Estimate	95% CI	Estimate	95% CI	Estimate	95% CI	Estimate	95% CI
Intercept	**5.476**	**(4.563**,** 6.39)**	**2.797**	**(2.171**,** 3.424)**	**3.154**	**(2.643**,** 3.667)**	**47.223**	**(19.481**,** 75.164)**	**138.589**	**(92.73**,** 184.768)**	**0.793**	**(0.699**,** 0.887)**
Child age at visit (month)	**0.034**	**(0.032**,** 0.036)**	**0.014**	**(0.013**,** 0.015)**	**0.017**	**(0.016**,** 0.018)**	**1.219**	**(1.159**,** 1.278)**	**0.356**	**(0.255**,** 0.455)**	**-0.002**	**(-0.002**,** -0.002)**
Child sex: female	**-0.103**	**(-0.175**,** -0.03)**	-0.026	(-0.075, 0.024)	-0.025	(-0.065, 0.015)	**-2.6**	**(-4.773**,** -0.435)**	0.556	(-3.033, 4.136)	0.007	(-0.001, 0.014)
Birth weight z-score *	**0.199**	**(0.155**,** 0.243)**	**0.124**	**(0.094**,** 0.154)**	**0.132**	**(0.107**,** 0.156)**	**9.122**	**(7.792**,** 10.469)**	**4.391**	**(2.192**,** 6.613)**	0.001	(-0.004, 0.005)
Gestational age	0.003	(-0.019, 0.026)	0.007	(-0.009, 0.022)	-0.004	(-0.017, 0.008)	0.103	(-0.58, 0.782)	0.166	(-0.963, 1.288)	0.001	(-0.002, 0.003)
Maternal age	0	(-0.007, 0.007)	-0.004	(-0.009, 0.001)	-0.001	(-0.005, 0.003)	-0.16	(-0.375, 0.055)	-0.262	(-0.616, 0.093)	0	(-0.001, 0.001)
Pre-pregnancy BMI												
Overweight	-0.01	(-0.1, 0.079)	-0.022	(-0.083, 0.04)	0.046	(-0.004, 0.096)	0.947	(-1.766, 3.663)	2.28	(-2.202, 6.769)	-0.003	(-0.013, 0.006)
Obese	-0.026	(-0.124, 0.071)	-0.046	(-0.112, 0.02)	-0.004	(-0.058, 0.05)	-1.465	(-4.382, 1.442)	-1.129	(-5.946, 3.668)	0	(-0.009, 0.01)
Race/ethnicity												
Non-Hispanic White	-0.03	(-0.162, 0.102)	0.006	(-0.084, 0.097)	-0.013	(-0.086, 0.061)	-0.292	(-4.263, 3.688)	-0.643	(-7.195, 5.914)	-0.004	(-0.018, 0.009)
Non-Hispanic Black	-0.159	(-0.383, 0.065)	0.114	(-0.04, 0.271)	0.06	(-0.067, 0.187)	1.516	(-5.258, 8.32)	1.011	(-10.129, 12.205)	**-0.031**	**(-0.054**,** -0.008)**
Asian	-0.005	(-0.179, 0.17)	-0.018	(-0.139, 0.102)	-0.033	(-0.131, 0.064)	-1.356	(-6.665, 3.956)	-2.875	(-11.678, 5.925)	-0.013	(-0.031, 0.005)
Other/Multiple race	-0.12	(-0.403, 0.163)	-0.047	(-0.247, 0.154)	-0.154	(-0.318, 0.011)	-2.44	(-11.548, 6.676)	-5.006	(-19.984, 9.992)	-0.013	(-0.042, 0.016)
Education												
Some college/Associate degree	-0.004	(-0.113, 0.105)	-0.021	(-0.095, 0.054)	-0.034	(-0.094, 0.027)	-1.893	(-5.15, 1.367)	-4.094	(-9.463, 1.279)	-0.007	(-0.018, 0.004)
Bachelor’s degree	0.059	(-0.077, 0.194)	-0.034	(-0.126, 0.059)	0.02	(-0.056, 0.096)	-0.769	(-4.848, 3.303)	-0.534	(-7.255, 6.181)	0.005	(-0.009, 0.019)
Post-graduate degree	0.063	(-0.088, 0.213)	-0.033	(-0.135, 0.07)	0.037	(-0.047, 0.121)	-0.185	(-4.734, 4.354)	0.148	(-7.355, 7.64)	0	(-0.016, 0.015)
Parity: Parous	0.064	(-0.023, 0.151)	**-0.065**	**(-0.125**,** -0.006)**	-0.036	(-0.084, 0.012)	-1.135	(-3.762, 1.487)	-2.369	(-6.701, 1.958)	0.004	(-0.004, 0.013)
Breastfeeding duration: more than 6 months	0.029	(-0.046, 0.105)	-0.021	(-0.073, 0.03)	**-0.046**	**(-0.088**,** -0.004)**	-2.03	(-4.293, 0.233)	**-4.892**	**(-8.63**,** -1.154)**	-0.002	(-0.009, 0.006)
Anthropometric z change *	**0.166**	**(0.137**,** 0.197)**	**0.126**	**(0.105**,** 0.147)**	**0.115**	**(0.098**,** 0.133)**	**8.266**	**(7.329**,** 9.233)**	**2.883**	**(1.335**,** 4.478)**	0.001	(-0.002, 0.004)

Notes: All models include time (age at visit in month) to adjust for child age; Bolded effect indicates statistical significance with a=0.05. TKV is the total kidney volume for right and left kidney. Length, width, depth are average of left and right kidney measurement. Adjusted TKV is the ratio of TKV and body surface area. Adjusted kidney length is the ratio of kidney length and child height. Birth weight z-scores are sex and gestational week dependent and were calculated through INTERGROWTH-21st tool. Anthropometric z change is the difference between birth weight z-score and weight-for-age z-score

We acknowledge the potential measurement error in the ultrasound-based measurements of kidney size [30]. Nevertheless, we do not consider this error to be differential or dependent across categories of the determinants. When comparing the baseline characteristics between the analytic sample and the sample without children’s kidney measurements, we observed differences in maternal characteristics. Mothers in the analytic sample were younger, had higher BMI, lower education levels, were more likely to be Hispanic, unemployed, and had public insurance (Table S7). Despite these differences, we were not concerned about selection bias, as it likely resulted from recruitment site differences rather than factors affecting child kidney size. However, it is important to acknowledge that the results may not be generalizable to all children. Lastly, it is important to emphasize that the goal of this study was not to establish causation, but to explore factors associated with kidney morphology in early childhood. These predictors may help inform future analyses of renal development by highlighting variables that need detailed investigation.

## Conclusion

In the NYU CHES study, we found that early childhood kidney size measures were consistently influenced by birth weight z-scores and changes in postnatal weight gain z-scores. Additionally, we observed racial differences and the influence of breastfeeding duration on kidney size. Future research should consider birth weight, postnatal growth patterns and key maternal characteristics to better understand kidney development in children.

## Supplementary Information

Below is the link to the electronic supplementary material.

Supplementary Material 1

## Data Availability

The data that has been used is confidential.

## Abbreviations

BMI: Body mass index
CHES: Children’s Health and Environment Study
CKD: Chronic kidney disease
DHQ II: Diet History Questionnaire II
EMR: Electronic medical records
GA: Gestational age
GDM: Gestational diabetes mellitus
HDP: Hypertension during pregnancy
HEI: The Healthy Eating Index-2015
ICC2: The Intraclass Correlation Coefficients 2
IQR: Interquartile ranges
LBW: Low birth weight
LMM: Linear mixed-effect models
NYU: The New York University
PTB: Preterm birth
SD: Standard deviations
SGA: Small for gestational age
TKV: Total kidney volume
WFA: Weight-for-age

## References

[1] Centers for Disease Control and Prevention. Chronic Kidney Disease in the United States. 2023. Chronic Kidney Disease in the United States, 2023. May 2023. Accessed January 20, 2025. https://www.cdc.gov/kidney-disease/media/pdfs/CKD-Factsheet-H.pdf

[2] Xie Y, Bowe B, Mokdad AH, et al. Analysis of the Global Burden of Disease study highlights the global, regional, and national trends of chronic kidney disease epidemiology from 1990 to 2016. Kidney Int. 2018;94(3):567–81. 10.1016/j.kint.2018.04.011. 30078514 10.1016/j.kint.2018.04.011

[3] Barker D, INFANT, MORTALITY, CHILDHOOD NUTRITION, AND ISCHAEMIC HEART DISEASE, IN ENGLAND AND WALES. Lancet. 1986;327(8489):1077–81. 10.1016/S0140-6736(86)91340-1. 10.1016/s0140-6736(86)91340-1 2871345

[4] Barker DJ, Osmond C, Golding J, Kuh D, Wadsworth ME. Growth in utero, blood pressure in childhood and adult life, and mortality from cardiovascular disease. BMJ. 1989;298(6673):564–7. 10.1136/bmj.298.6673.564. 2495113 10.1136/bmj.298.6673.564 PMC1835925

[5] Brenner BM, Chertow GM. Congenital Oligonephropathy and the Etiology of Adult Hypertension and Progressive Renal Injury. Am J Kidney Dis. 1994;23(2):171–5. 10.1016/S0272-6386(12)80967-X. 8311070

[6] White SL, Perkovic V, Cass A, et al. Is Low Birth Weight an Antecedent of CKD in Later Life? A Systematic Review of Observational Studies. Am J Kidney Dis. 2009;54(2):248–61. 10.1053/j.ajkd.2008.12.042. 19339091 10.1053/j.ajkd.2008.12.042

[7] Brophy P. Maternal determinants of renal mass and function in the fetus and neonate. Semin Fetal Neonatal Med. 2017;22(2):67–70. 10.1016/j.siny.2017.01.004. 28347404 10.1016/j.siny.2017.01.004

[8] Iyengar A, Nesargi S, George A, Sinha N, Selvam S, Luyckx VA. Are low birth weight neonates at risk for suboptimal renal growth and function during infancy? BMC Nephrol. 2016;17(1):100. 10.1186/s12882-016-0314-7. 27460896 10.1186/s12882-016-0314-7 PMC4962347

[9] Kandasamy Y, Rudd D, Lumbers ER, Smith R. An evaluation of preterm kidney size and function over the first two years of life. Pediatr Nephrol. 2020;35(8):1477–82. 10.1007/s00467-020-04554-y. 32297001 10.1007/s00467-020-04554-y PMC7316836

[10] Bakker H, Gaillard R, Franco OH, et al. Fetal and Infant Growth Patterns and Kidney Function at School Age. J Am Soc Nephrol. 2014;25(11):2607–15. 10.1681/ASN.2013091003. 24812164 10.1681/ASN.2013091003 PMC4214527

[11] Geelhoed JJM, Verburg BO, Nauta J, et al. Tracking and Determinants of Kidney Size From Fetal Life Until the Age of 2 Years: The Generation R Study. Am J Kidney Dis. 2009;53(2):248–58. 10.1053/j.ajkd.2008.07.030. 18848377 10.1053/j.ajkd.2008.07.030

[12] Geelhoed JJM, Taal HR, Steegers EAP, et al. Kidney growth curves in healthy children from the third trimester of pregnancy until the age of two years. The Generation R Study. Pediatr Nephrol. 2010;25(2):289–98. 10.1007/s00467-009-1335-2. 19898876 10.1007/s00467-009-1335-2 PMC7811527

[13] Alam AT, Ijaz I, Mukhtar MU, et al. Comparison of renal growth in breast fed and artificial fed infants: a cross-sectional study. BMC Res Notes. 2023;16(1):143. 10.1186/s13104-023-06368-1. 37430332 10.1186/s13104-023-06368-1 PMC10334511

[14] Parada-Ricart E, Ferre N, Luque V, et al. Effect of Protein Intake Early in Life on Kidney Volume and Blood Pressure at 11 Years of Age. Nutrients. 2023;15(4):874. 10.3390/nu15040874. 36839233 10.3390/nu15040874 PMC9961192

[15] Schmidt IM, Damgaard IN, Boisen KA, et al. Increased kidney growth in formula-fed versus breast-fed healthy infants. Pediatr Nephrol. 2004;19(10). 10.1007/s00467-004-1567-0. 10.1007/s00467-004-1567-0 15309602

[16] Miliku K, Voortman T, Bakker H, Hofman A, Franco OH, Jaddoe VWV. Infant Breastfeeding and Kidney Function in School-Aged Children. Am J Kidney Dis. 2015;66(3):421–8. 10.1053/j.ajkd.2014.12.018. 25747235 10.1053/j.ajkd.2014.12.018

[17] Escribano J, Luque V, Ferre N, et al. Increased protein intake augments kidney volume and function in healthy infants. Kidney Int. 2011;79(7):783–90. 10.1038/ki.2010.499. 21191362 10.1038/ki.2010.499

[18] Lee YQ, Collins CE, Gordon A, Rae KM, Pringle KG. The Relationship between Maternal Nutrition during Pregnancy and Offspring Kidney Structure and Function in Humans: A Systematic Review. Nutrients. 2018;10(2):241. 10.3390/nu10020241. 29466283 10.3390/nu10020241 PMC5852817

[19] Hsu CW, Yamamoto KT, Henry RK, De Roos AJ, Flynn JT. Prenatal Risk Factors for Childhood CKD. J Am Soc Nephrol. 2014;25(9):2105–11. 10.1681/ASN.2013060582. 24744441 10.1681/ASN.2013060582 PMC4147970

[20] Trasande L, Ghassabian A, Kahn LG, et al. The NYU Children’s Health and Environment Study. Eur J Epidemiol. 2020;35(3):305–20. 10.1007/s10654-020-00623-6. 32212050 10.1007/s10654-020-00623-6 PMC7154015

[21] The Global Health Network. INTERGROWTH-21st Standards and Tools. December 11. 2024. Accessed January 6, 2025. https://intergrowth21.com/intergrowth-21st-applications-calculators

[22] Hafen R, Hathaway J. ki-tools/growthstandards. Accessed January 6, 2025. https://ki-tools.github.io/growthstandards/

[23] Elmrayed S, Dai S, Lodha A, Kumar M, Fenton TR. Preterm growth assessment: the latest findings on age correction. J Perinatol. 2025;45(5):607–15. 10.1038/s41372-024-02202-z. 39820765 10.1038/s41372-024-02202-z PMC12221983

[24] Subar AF, Thompson FE, Kipnis V, et al. Comparative Validation of the Block, Willett, and National Cancer Institute Food Frequency Questionnaires. Am J Epidemiol. 2001;154(12):1089–99. 10.1093/aje/154.12.1089. 11744511 10.1093/aje/154.12.1089

[25] NIH. Development of the DHQ II and C-DHQ II Nutrition & Food Group Database. August 19. 2024. Accessed January 6, 2025. https://epi.grants.cancer.gov/dhq2/database/

[26] Reedy J, Lerman JL, Krebs-Smith SM, et al. Evaluation of the Healthy Eating Index-2015. J Acad Nutr Diet. 2018;118(9):1622–33. 10.1016/j.jand.2018.05.019. 30146073 10.1016/j.jand.2018.05.019 PMC6718954

[27] Bakker H, Kooijman MN, van der Heijden AJ, et al. Kidney size and function in a multi-ethnic population-based cohort of school-age children. Pediatr Nephrol. 2014;29(9):1589–98. 10.1007/s00467-014-2793-8. 24599444 10.1007/s00467-014-2793-8

[28] Eneanya ND, Yang W, Reese PP. Reconsidering the Consequences of Using Race to Estimate Kidney Function. JAMA. 2019;322(2):113. 10.1001/jama.2019.5774. 31169890 10.1001/jama.2019.5774

[29] Fontanarosa PB, Bauchner H. Race, Ancestry, and Medical Research. JAMA. 2018;320(15):1539. 10.1001/jama.2018.14438. 30264148 10.1001/jama.2018.14438

[30] Al Salmi I, Hajriy A. Mahmood, Hannawi, Suad. Ultrasound Measurement and Kidney Development a Mini-Review for Nephrologists. Saudi Journal of Kidney Diseases and Transplantation. 2021;32(1):p 174–182,. 10.4103/1319-2442.318520. 10.4103/1319-2442.318520 34145128

