# Supplementary material for: Maternal and fetal determinants on kidney size in early childhood: insights from a New York City cohort
Source: BMC Nephrol. 2026 Apr 14;27:331. doi: 10.1186/s12882-026-04913-1 (PMC13202755; doi:10.1186/s12882-026-04913-1)
Supplement: Supplementary file 1 — Supplementary Material 1 [file 12882_2026_4913_MOESM1_ESM.pdf]

Maternal and fetal determinants on kidney size in early childhood: Insights from a New York City cohort

Supplementary Materials

Table S1. Estimates from unadjusted linear mixed effect model with adjustment for age at visit for each of the kidney size outcome, excluding those who smoke during pregnancy.*

	Average kidney length		Average kidney width		Average kidney depth		TKV		Adjusted TKV		Adjusted kidney length	
Exposure	Coefficient	90% CI	Coefficient	90% CI	Coefficient	90% CI	Coefficient	90% CI	Coefficient	90% CI	Coefficient	90% CI
Maternal age	0.001	(-0.01, 0.01)	**-0.007**	**(-0.01, 0)**	-0.001	(-0.01, 0)	**-0.236**	**(-0.46, -0.01)**	**-0.316**	**(-0.63, -0.01)**	0	(0, 0)
Pre-pregnancy BMI category												
Overweight	0.041	(-0.05, 0.13)	0.01	(-0.05, 0.07)	**0.059**	**(0.01, 0.11)**	**3.263**	**(0.21, 6.31)**	3.966	(-0.27, 8.2)	0.001	(-0.01, 0.01)
Obese	**0.104**	**(0.01, 0.2)**	0.047	(-0.02, 0.11)	**0.066**	**(0.01, 0.12)**	**4.846**	**(1.68, 8.01)**	2.205	(-2.18, 6.59)	0.005	(0, 0.01)
Race/ethnicity												
Non-Hispanic White	-0.071	(-0.16, 0.02)	-0.056	(-0.12, 0.01)	-0.035	(-0.09, 0.02)	**-3.843**	**(-7.11, -0.57)**	-2.155	(-6.65, 2.34)	-0.003	(-0.01, 0.01)
Non-Hispanic Black	-0.158	(-0.36, 0.04)	0.038	(-0.1, 0.18)	0.004	(-0.12, 0.12)	-1.549	(-8.5, 5.4)	-0.138	(-10.25, 9.98)	**-0.026**	**(-0.05, -0.01)**
Asian	-0.082	(-0.23, 0.06)	**-0.112**	**(-0.21, -0.01)**	-0.066	(-0.15, 0.02)	**-6.87**	**(-11.95, -1.79)**	-4.671	(-11.79, 2.44)	-0.012	(-0.03, 0)
Other/Multiple race	-0.112	(-0.38, 0.16)	-0.1	(-0.29, 0.09)	-0.121	(-0.28, 0.04)	-5.816	(-15.5, 3.87)	-6.861	(-20.92, 7.2)	-0.016	(-0.04, 0.01)
Maritial status												
Divorced/separated/single/widowed	0.041	(-0.07, 0.15)	0.042	(-0.03, 0.12)	0.044	(-0.02, 0.11)	2.498	(-1.3, 6.29)	5.073	(-0.15, 10.29)	-0.002	(-0.01, 0.01)
Education												
Some college but no degree/Associate degree	-0.047	(-0.15, 0.06)	-0.005	(-0.08, 0.07)	-0.034	(-0.09, 0.03)	-1.701	(-5.39, 1.99)	-2.272	(-7.39, 2.84)	-0.008	(-0.02, 0)
Bachelor's degree	-0.052	(-0.15, 0.05)	-0.049	(-0.12, 0.02)	-0.034	(-0.09, 0.03)	-3.463	(-7.06, 0.14)	-2.538	(-7.58, 2.5)	-0.004	(-0.01, 0.01)
Post-graduate degree	-0.073	(-0.17, 0.03)	**-0.071**	**(-0.14, 0)**	-0.034	(-0.09, 0.03)	**-5.128**	**(-8.7, -1.56)**	-3.824	(-8.75, 1.1)	-0.009	(-0.02, 0)
Employed	-0.031	(-0.1, 0.04)	-0.03	(-0.08, 0.02)	0.021	(-0.02, 0.06)	-1.915	(-4.44, 0.61)	-2.784	(-6.28, 0.71)	-0.005	(-0.01, 0)

Insurance: Private	0.004	(-0.07, 0.08)	-0.035	(-0.09, 0.02)	-0.013	(-0.06, 0.03)	-2.096	(-4.84, 0.65)	-1.54	(-5.33, 2.25)	-0.002	(-0.01, 0.01)
Parity: Parous	0.068	(-0.01, 0.14)	**-0.053**	**(-0.1, 0)**	-0.014	(-0.06, 0.03)	0.097	(-2.56, 2.76)	-1.84	(-5.53, 1.85)	0.005	(0, 0.01)
Alcohol during pregnancy	-0.046	(-0.15, 0.05)	0.046	(-0.02, 0.11)	0.01	(-0.05, 0.07)	0.204	(-3.34, 3.75)	1.681	(-3.17, 6.53)	-0.001	(-0.01, 0.01)
GDM	-0.025	(-0.11, 0.06)	0.005	(-0.05, 0.06)	0.024	(-0.02, 0.07)	0.177	(-2.67, 3.02)	1.872	(-2.05, 5.8)	0	(-0.01, 0.01)
Anxiety	-0.06	(-0.19, 0.07)	-0.017	(-0.11, 0.08)	-0.026	(-0.1, 0.05)	-1.066	(-5.7, 3.56)	3.405	(-3, 9.81)	0.006	(-0.01, 0.02)
Depression	0.027	(-0.1, 0.15)	-0.003	(-0.09, 0.08)	0.016	(-0.06, 0.09)	0.902	(-3.29, 5.1)	2.568	(-3.21, 8.35)	0.006	(-0.01, 0.02)
HDP	-0.042	(-0.14, 0.06)	-0.004	(-0.07, 0.06)	-0.014	(-0.07, 0.04)	-0.403	(-3.75, 2.94)	-1.054	(-5.68, 3.57)	-0.003	(-0.01, 0.01)
**Fetal determinants**												
Child sex: female	**-0.106**	**(-0.18, -0.03)**	-0.026	(-0.08, 0.02)	-0.022	(-0.06, 0.02)	**-2.789**	**(-5.31, -0.26)**	0.352	(-3.15, 3.85)	0.006	(0, 0.01)
Gestational age at birth	0.017	(-0.01, 0.04)	**0.016**	**(0, 0.03)**	0.005	(-0.01, 0.02)	**0.786**	**(0.02, 1.55)**	0.609	(-0.46, 1.68)	0.001	(0, 0)
Birth weight z-score *	**0.109**	**(0.07, 0.15)**	**0.048**	**(0.02, 0.07)**	**0.063**	**(0.04, 0.08)**	**4.697**	**(3.38, 6.01)**	**2.452**	**(0.58, 4.32)**	0.001	(0, 0.01)
LBW	**-0.17**	**(-0.31, -0.03)**	**-0.18**	**(-0.28, -0.08)**	**-0.107**	**(-0.19, -0.02)**	**-9.893**	**(-14.85, -4.94)**	-6.605	(-13.63, 0.42)	-0.001	(-0.01, 0.01)
PTB	-0.004	(-0.14, 0.14)	**-0.102**	**(-0.2, -0.01)**	-0.031	(-0.11, 0.05)	-2.967	(-7.88, 1.94)	-2.846	(-9.72, 4.02)	-0.002	(-0.02, 0.01)
SGA	**-0.208**	**(-0.37, -0.04)**	**-0.162**	**(-0.27, -0.05)**	**-0.15**	**(-0.25, -0.05)**	**-11.686**	**(-17.4, -5.97)**	-6.79	(-15.04, 1.45)	0.004	(-0.01, 0.02)
**Diet during pregnancy ***												
HEI score	0.003	(0, 0.01)	-0.001	(0, 0)	0	(0, 0)	0.024	(-0.14, 0.18)	0.017	(-0.2, 0.24)	0	(0, 0)
Energy (log)	0.008	(-0.05, 0.06)	-0.018	(-0.06, 0.02)	-0.006	(-0.04, 0.03)	0.051	(-1.86, 1.97)	-0.891	(-3.5, 1.72)	-0.001	(-0.01, 0)
Protein (log)	0.011	(-0.03, 0.06)	-0.012	(-0.04, 0.02)	-0.006	(-0.03, 0.02)	0.088	(-1.49, 1.67)	-0.511	(-2.66, 1.63)	0	(0, 0)
Retinol (log)	0.014	(-0.03, 0.06)	-0.014	(-0.04, 0.02)	-0.009	(-0.03, 0.02)	0.093	(-1.39, 1.57)	-0.685	(-2.69, 1.32)	-0.001	(0, 0)
Folate (log)	0.005	(-0.04, 0.06)	-0.015	(-0.05, 0.02)	-0.006	(-0.03, 0.02)	-0.024	(-1.74, 1.69)	-0.315	(-2.65, 2.02)	0	(0, 0)
Sodium (log)	0.005	(-0.04, 0.05)	-0.011	(-0.04, 0.02)	-0.005	(-0.03, 0.02)	0.099	(-1.52, 1.72)	-0.655	(-2.86, 1.55)	0	(0, 0)
Potassium (log)	0.007	(-0.05, 0.06)	-0.025	(-0.06, 0.01)	-0.005	(-0.04, 0.03)	0.189	(-1.74, 2.11)	-0.864	(-3.48, 1.75)	0	(-0.01, 0.01)
**Postnatal growth**												
Weight for age z-score	**0.171**	**(0.14, 0.2)**	**0.124**	**(0.11, 0.14)**	**0.116**	**(0.1, 0.13)**	**8.356**	**(7.5, 9.21)**	**3.304**	**(1.89, 4.72)**	0.002	(0, 0)
Anthropometric z change *	**0.103**	**(0.08, 0.13)**	**0.088**	**(0.07, 0.11)**	**0.072**	**(0.06, 0.09)**	**5.265**	**(4.37, 6.16)**	**1.569**	**(0.21, 2.92)**	0.001	(0, 0)

Breastfeeding duration: more than 6 months	-0.01	(-0.09, 0.07)	**-0.055**	**(-0.11, 0)**	**-0.067**	**(-0.11, -0.02)**	**-4.045**	**(-6.67, -1.41)**	**-6.333**	**(-9.95, -2.72)**	-0.003	(-0.01, 0)

Notes: All models include time (age at visit in month) to adjust for child age; Bolded effect indicates statistically significance with a=0.1.

TKV is the total kidney volume for right and left kidney. Length, width, depth are average of left and right kidney measurement. Adjusted TKV is the ratio of tkv and body surface area. Adjusted kidney length is the ratio of kidney length and child height. Birth weight z-scores are sex and gestational week dependent and were calculated through INTERGROWTH-21st tool. All diet exposures are natural log-transformed. Anthropometric z change is the difference of birthweight z-score and weight-for-age z-score.

Table S2: Model 2 - Adjusted LMMs for kidney outcomes (adjusted for maternal, fetal determinants, breastfeeding duration, and z-score difference) with quadratic term for child age.

	Average Kidney Length (N=787)		Average Kidney Width (N=774)		Average kidney depth (N=775)		TKV (N=721)		Adjusted TKV (N=716)		Adjusted Kidney Length (N=746)	
	Estimate	95% CI	Estimate	95% CI	Estimate	95% CI	Estimate	95% CI	Estimate	95% CI	Estimate	95% CI
Intercept	**5.184**	**(4.273, 6.096)**	**2.679**	**(2.051, 3.308)**	**3.028**	**(2.516, 3.541)**	**44.808**	**(16.974, 72.812)**	**138.119**	**(92.036, 184.471)**	**0.826**	**(0.732, 0.92)**
Child age at visit (month)	**0.052**	**(0.046, 0.058)**	**0.021**	**(0.017, 0.026)**	**0.025**	**(0.021, 0.029)**	**1.398**	**(1.189, 1.607)**	**0.396**	**(0.024, 0.768)**	**-0.004**	**(-0.005, -0.004)**
Child age at visit (month) ^2	**0**	**(0, 0)**	**0**	**(0, 0)**	**0**	**(0, 0)**	-0.002	(-0.005, 0)	-0.001	(-0.005, 0.004)	**0**	**(0, 0)**
Child sex: female	**-0.098**	**(-0.17, -0.026)**	-0.024	(-0.073, 0.025)	-0.023	(-0.063, 0.016)	**-2.553**	**(-4.722, -0.391)**	0.566	(-3.023, 4.147)	0.006	(-0.002, 0.013)
Birth weight z-score *	**0.194**	**(0.151, 0.238)**	**0.123**	**(0.093, 0.153)**	**0.13**	**(0.106, 0.155)**	**9.107**	**(7.779, 10.451)**	**4.384**	**(2.184, 6.606)**	0.001	(-0.003, 0.006)
Gestational age	0.004	(-0.018, 0.026)	0.007	(-0.009, 0.022)	-0.004	(-0.017, 0.008)	0.094	(-0.588, 0.772)	0.163	(-0.966, 1.285)	0.001	(-0.002, 0.003)
Maternal age	0.001	(-0.006, 0.008)	-0.004	(-0.009, 0.001)	-0.001	(-0.005, 0.003)	-0.152	(-0.366, 0.063)	-0.26	(-0.615, 0.095)	0	(-0.001, 0.001)
Pre-pregnancy BMI												
Overweight	-0.013	(-0.102, 0.077)	-0.023	(-0.084, 0.039)	0.045	(-0.005, 0.095)	0.963	(-1.745, 3.674)	2.283	(-2.199, 6.771)	-0.004	(-0.013, 0.006)
Obese	-0.029	(-0.126, 0.067)	-0.048	(-0.114, 0.018)	-0.006	(-0.059, 0.048)	-1.494	(-4.405, 1.408)	-1.137	(-5.953, 3.66)	0.001	(-0.009, 0.01)
Race/ethnicity												
Non-Hispanic White	-0.037	(-0.168, 0.094)	0.004	(-0.086, 0.094)	-0.015	(-0.088, 0.058)	-0.325	(-4.289, 3.647)	-0.652	(-7.204, 5.904)	-0.004	(-0.017, 0.01)
Non-Hispanic Black	-0.165	(-0.387, 0.057)	0.113	(-0.041, 0.269)	0.058	(-0.068, 0.185)	1.439	(-5.324, 8.23)	0.996	(-10.145, 12.188)	**-0.03**	**(-0.052, -0.007)**
Asian	-0.022	(-0.195, 0.152)	-0.026	(-0.146, 0.094)	-0.041	(-0.139, 0.056)	-1.557	(-6.861, 3.75)	-2.918	(-11.73, 5.888)	-0.01	(-0.028, 0.008)
Other/Multiple race	-0.132	(-0.413, 0.149)	-0.051	(-0.251, 0.149)	-0.158	(-0.321, 0.006)	-2.444	(-11.535, 6.655)	-5.005	(-19.981, 9.99)	-0.012	(-0.041, 0.017)
Education												
Some college/Associate degree	-0.006	(-0.115, 0.103)	-0.022	(-0.096, 0.052)	-0.036	(-0.096, 0.024)	-1.933	(-5.185, 1.32)	-4.097	(-9.465, 1.275)	-0.007	(-0.018, 0.004)
Bachelor's degree	0.066	(-0.069, 0.201)	-0.031	(-0.123, 0.062)	0.023	(-0.052, 0.099)	-0.73	(-4.802, 3.334)	-0.517	(-7.238, 6.199)	0.004	(-0.01, 0.018)
Post-graduate degree	0.071	(-0.079, 0.22)	-0.029	(-0.132, 0.073)	0.041	(-0.043, 0.124)	-0.149	(-4.689, 4.382)	0.158	(-7.344, 7.65)	-0.001	(-0.016, 0.014)

Parity: Parous	0.067	(-0.02, 0.153)	**-0.064**	**(-0.123, -0.006)**	-0.035	(-0.083, 0.013)	-1.14	(-3.762, 1.477)	-2.364	(-6.695, 1.962)	0.004	(-0.005, 0.013)
Breastfeeding duration: more than 6 months	0.014	(-0.061, 0.089)	-0.027	(-0.079, 0.024)	**-0.053**	**(-0.094, -0.011)**	-2.166	(-4.429, 0.098)	**-4.922**	**(-8.67, -1.175)**	0	(-0.008, 0.008)
Anthropometric z change *	**0.162**	**(0.133, 0.192)**	**0.125**	**(0.104, 0.146)**	**0.114**	**(0.097, 0.131)**	**8.24**	**(7.303, 9.206)**	**2.874**	**(1.321, 4.47)**	0.002	(-0.001, 0.005)

Notes: All models include time (age at visit in month) to adjust for child age; Bolded effect indicates statistically significance with a=0.05.

TKV is the total kidney volume for right and left kidney. Length, width, depth are average of left and right kidney measurement. Adjusted TKV is the ratio of tkv and body surface area. Adjusted kidney length is the ratio of kidney length and child height. Birth weight z-scores are sex and gestational week dependent and were calculated through INTERGROWTH-21st tool. Anthropometric z change is the difference of birthweight z-score and weight-for-age z-score.

Table S3: Model 2 - Adjusted LMMs for kidney outcomes (adjusted for maternal, fetal determinants, breastfeeding duration, and z-score difference) using sample excluding PTB infants.

	Average Kidney Length (N=731)		Average Kidney Width (N=719)		Average kidney depth (N=720)		TKV (N=670)		Adjusted TKV (N=666)		Adjusted Kidney Length (N=694)	
	Estimate	95% CI	Estimate	95% CI	Estimate	95% CI	Estimate	95% CI	Estimate	95% CI	Estimate	95% CI
Intercept	**5.339**	**(3.918, 6.767)**	**3.481**	**(2.499, 4.464)**	**3.276**	**(2.488, 4.067)**	**52.677**	**(9.86, 95.837)**	**135.02**	**(64.465, 206.159)**	**0.791**	**(0.649, 0.934)**
Child age at visit (month)	**0.034**	**(0.032, 0.036)**	**0.014**	**(0.013, 0.015)**	**0.018**	**(0.016, 0.019)**	**1.219**	**(1.156, 1.28)**	**0.371**	**(0.265, 0.473)**	**-0.002**	**(-0.002, -0.002)**
Child sex: female	**-0.114**	**(-0.189, -0.039)**	-0.035	(-0.087, 0.017)	-0.023	(-0.065, 0.018)	**-2.723**	**(-4.988, -0.464)**	0.523	(-3.214, 4.253)	0.006	(-0.002, 0.014)
Birth weight z-score *	**0.216**	**(0.17, 0.262)**	**0.117**	**(0.085, 0.149)**	**0.124**	**(0.098, 0.15)**	**8.881**	**(7.488, 10.293)**	**3.994**	**(1.693, 6.321)**	0.002	(-0.002, 0.007)
Gestational age	0.007	(-0.029, 0.042)	-0.01	(-0.034, 0.014)	-0.008	(-0.027, 0.012)	-0.055	(-1.119, 1)	0.201	(-1.553, 1.94)	0.001	(-0.003, 0.004)
Maternal age	0.001	(-0.007, 0.008)	-0.004	(-0.009, 0.001)	-0.001	(-0.005, 0.003)	-0.115	(-0.344, 0.113)	-0.172	(-0.549, 0.205)	0	(-0.001, 0.001)
Pre-pregnancy BMI												
Overweight	0.001	(-0.093, 0.095)	-0.032	(-0.097, 0.033)	0.046	(-0.006, 0.098)	0.943	(-1.889, 3.777)	2.536	(-2.139, 7.213)	-0.001	(-0.011, 0.008)
Obese	-0.04	(-0.143, 0.062)	-0.058	(-0.128, 0.013)	-0.011	(-0.068, 0.045)	-1.897	(-4.96, 1.152)	-1.448	(-6.504, 3.582)	0	(-0.01, 0.011)
Race/ethnicity												
Non-Hispanic White	-0.008	(-0.148, 0.134)	-0.007	(-0.104, 0.09)	-0.019	(-0.098, 0.059)	-0.319	(-4.578, 3.95)	-0.507	(-7.528, 6.518)	0.001	(-0.013, 0.015)
Non-Hispanic Black	-0.241	(-0.484, 0.001)	0.128	(-0.039, 0.296)	0.072	(-0.062, 0.207)	0.947	(-6.27, 8.2)	0.375	(-11.478, 12.296)	**-0.042**	**(-0.066, -0.018)**
Asian	0.024	(-0.162, 0.211)	-0.034	(-0.164, 0.096)	-0.016	(-0.12, 0.089)	-0.721	(-6.431, 4.997)	-1.721	(-11.187, 7.746)	-0.009	(-0.027, 0.011)
Other/Multiple race	-0.183	(-0.498, 0.132)	-0.042	(-0.27, 0.187)	-0.134	(-0.318, 0.051)	-1.459	(-11.877, 8.989)	-3.456	(-20.562, 13.706)	-0.022	(-0.054, 0.01)
Education												
Some college/Associate degree	0.025	(-0.09, 0.139)	-0.029	(-0.107, 0.05)	-0.018	(-0.081, 0.045)	-0.834	(-4.24, 2.57)	-2.591	(-8.199, 3.015)	-0.004	(-0.015, 0.007)
Bachelor's degree	0.029	(-0.114, 0.172)	-0.032	(-0.131, 0.066)	0.02	(-0.06, 0.099)	-1.249	(-5.547, 3.035)	-1.128	(-8.201, 5.929)	-0.001	(-0.015, 0.013)
Post-graduate degree	0.034	(-0.125, 0.193)	-0.024	(-0.134, 0.085)	0.037	(-0.051, 0.126)	-0.168	(-4.989, 4.641)	0.133	(-7.814, 8.063)	-0.004	(-0.02, 0.012)
Parity: Parous	0.063	(-0.028, 0.154)	**-0.077**	**(-0.14, -0.015)**	-0.041	(-0.091, 0.009)	-1.324	(-4.077, 1.421)	-3.102	(-7.637, 1.426)	0.002	(-0.007, 0.012)

Breastfeeding duration: more than 6 months	0.014	(-0.065, 0.093)	-0.027	(-0.082, 0.027)	-0.059	(-0.103, -0.016)	**-2.554**	**(-4.917, -0.189)**	**-5.852**	**(-9.752, -1.95)**	-0.002	(-0.01, 0.006)
Anthropometric z change *	**0.168**	**(0.136, 0.199)**	**0.122**	**(0.1, 0.145)**	**0.113**	**(0.095, 0.131)**	**8.145**	**(7.169, 9.156)**	**2.641**	**(1.032, 4.303)**	0.002	(-0.002, 0.005)

Notes: All models include time (age at visit in month) to adjust for child age; Bolded effect indicates statistically significance with a=0.05.

TKV is the total kidney volume for right and left kidney. Length, width, depth are average of left and right kidney measurement. Adjusted TKV is the ratio of tkv and body surface area. Adjusted kidney length is the ratio of kidney length and child height. Birth weight z-scores are sex and gestational week dependent and were calculated through INTERGROWTH-21st tool. Anthropometric z change is the difference of birthweight z-score and weight-for-age z-score.

Table S4: Model 2 - Adjusted LMMs for kidney outcomes (adjusted for maternal, fetal determinants, breastfeeding duration, and z-score difference) using sample excluding LBW infants.

	Average Kidney Length (N=735)		Average Kidney Width (N=723)		Average kidney depth (N=724)		TKV (N=673)		Adjusted TKV (N=668)		Adjusted Kidney Length (N=696)	
	Estimate	95% CI	Estimate	95% CI	Estimate	95% CI	Estimate	95% CI	Estimate	95% CI	Estimate	95% CI
Intercept	**5.223**	**(3.978, 6.472)**	**3.218**	**(2.357, 4.08)**	**3.343**	**(2.651, 4.035)**	**52.233**	**(14.813, 89.887)**	**136.4**	**(74.135, 199.091)**	**0.749**	**(0.624, 0.875)**
Child age at visit (month)	**0.034**	**(0.032, 0.036)**	**0.014**	**(0.013, 0.016)**	**0.017**	**(0.016, 0.019)**	**1.229**	**(1.166, 1.291)**	**0.375**	**(0.268, 0.479)**	**-0.002**	**(-0.002, -0.002)**
Child sex: female	**-0.099**	**(-0.174, -0.024)**	-0.027	(-0.079, 0.024)	-0.019	(-0.061, 0.022)	-2.232	(-4.501, 0.028)	1.06	(-2.7, 4.81)	0.007	(-0.001, 0.014)
Birth weight z-score *	**0.213**	**(0.163, 0.262)**	**0.118**	**(0.084, 0.152)**	**0.132**	**(0.105, 0.159)**	**9.113**	**(7.625, 10.623)**	**4.246**	**(1.778, 6.742)**	0.002	(-0.003, 0.007)
Gestational age	0.009	(-0.021, 0.04)	-0.004	(-0.025, 0.017)	-0.009	(-0.026, 0.008)	-0.045	(-0.974, 0.877)	0.193	(-1.352, 1.728)	0.002	(-0.001, 0.005)
Maternal age	-0.001	(-0.008, 0.007)	-0.004	(-0.009, 0.001)	-0.002	(-0.006, 0.002)	-0.141	(-0.367, 0.086)	-0.247	(-0.623, 0.129)	0	(-0.001, 0.001)
Pre-pregnancy BMI												
Overweight	0.002	(-0.093, 0.097)	-0.041	(-0.106, 0.025)	0.039	(-0.013, 0.092)	0.71	(-2.148, 3.573)	2.022	(-2.715, 6.767)	0	(-0.01, 0.009)
Obese	-0.025	(-0.128, 0.077)	-0.061	(-0.132, 0.009)	-0.012	(-0.069, 0.044)	-1.776	(-4.853, 1.289)	-1.353	(-6.451, 3.721)	0.002	(-0.008, 0.013)
Race/ethnicity												
Non-Hispanic White	0.001	(-0.138, 0.14)	-0.005	(-0.101, 0.091)	-0.02	(-0.097, 0.058)	-0.189	(-4.392, 4.029)	-0.455	(-7.406, 6.506)	0.001	(-0.013, 0.015)
Non-Hispanic Black	-0.229	(-0.484, 0.024)	0.159	(-0.014, 0.335)	0.106	(-0.034, 0.247)	2.254	(-5.283, 9.83)	3.367	(-9.057, 15.859)	**-0.039**	**(-0.065, -0.014)**
Asian	0.031	(-0.155, 0.218)	-0.056	(-0.186, 0.074)	-0.029	(-0.133, 0.075)	-1.587	(-7.304, 4.139)	-3.022	(-12.534, 6.49)	-0.007	(-0.026, 0.012)
Other/Multiple race	-0.211	(-0.544, 0.123)	-0.063	(-0.307, 0.18)	-0.163	(-0.36, 0.035)	-2.815	(-14.163, 8.558)	-5.386	(-24.053, 13.332)	-0.025	(-0.059, 0.009)
Education												
Some college/Associate degree	0.008	(-0.107, 0.123)	-0.025	(-0.103, 0.054)	-0.023	(-0.086, 0.04)	-1.279	(-4.695, 2.136)	-3.142	(-8.788, 2.501)	-0.006	(-0.017, 0.006)
Bachelor's degree	0.029	(-0.113, 0.17)	-0.023	(-0.121, 0.074)	0.027	(-0.051, 0.106)	-0.824	(-5.09, 3.434)	-0.415	(-7.46, 6.622)	0	(-0.014, 0.014)
Post-graduate degree	0.037	(-0.122, 0.196)	-0.026	(-0.135, 0.083)	0.043	(-0.045, 0.131)	-0.006	(-4.839, 4.812)	0.489	(-7.503, 8.459)	-0.002	(-0.018, 0.014)
Parity: Parous	0.075	(-0.015, 0.166)	**-0.076**	**(-0.138, -0.014)**	-0.041	(-0.091, 0.009)	-1.215	(-3.961, 1.524)	-2.486	(-7.023, 2.046)	0.005	(-0.004, 0.014)
Breastfeeding duration: more than 6 months	0.036	(-0.043, 0.115)	-0.029	(-0.084, 0.025)	**-0.052**	**(-0.095, -0.008)**	-2.301	(-4.663, 0.062)	**-5.123**	**(-9.035, -1.208)**	-0.001	(-0.009, 0.007)
Anthropometric z change *	**0.171**	**(0.139, 0.203)**	**0.123**	**(0.101, 0.146)**	**0.115**	**(0.097, 0.133)**	**8.26**	**(7.286, 9.266)**	**2.87**	**(1.259, 4.531)**	0.002	(-0.001, 0.005)

Notes: All models include time (age at visit in month) to adjust for child age; Bolded effect indicates statistically significance with a=0.05.

TKV is the total kidney volume for right and left kidney. Length, width, depth are average of left and right kidney measurement. Adjusted TKV is the ratio of tkv and body surface area. Adjusted kidney length is the ratio of kidney length and child height. Birth weight z-scores are sex and gestational week dependent and were calculated through INTERGROWTH-21st tool. Anthropometric z change is the difference of birthweight z-score and weight-for-age z-score.

Table S5: Model 2 - Adjusted LMMs for kidney outcomes (adjusted for maternal, fetal determinants, breastfeeding duration, and z-score difference) using sample excluding SGA infants.

	Average Kidney Length (N=750)		Average Kidney Width (N=738)		Average kidney depth (N=739)		TKV (N=687)		Adjusted TKV (N=682)		Adjusted Kidney Length (N=711)	
	Estimate	95% CI	Estimate	95% CI	Estimate	95% CI	Estimate	95% CI	Estimate	95% CI	Estimate	95% CI
Intercept	**5.456**	**(4.492, 6.423)**	**2.86**	**(2.195, 3.527)**	**3.263**	**(2.724, 3.803)**	**50.778**	**(21.373, 80.421)**	**146.719**	**(97.988, 195.843)**	**0.804**	**(0.706, 0.903)**
Child age at visit (month)	**0.035**	**(0.033, 0.036)**	**0.014**	**(0.012, 0.015)**	**0.017**	**(0.016, 0.018)**	**1.227**	**(1.165, 1.287)**	**0.362**	**(0.257, 0.463)**	**-0.002**	**(-0.002, -0.002)**
Child sex: female	**-0.101**	**(-0.175, -0.026)**	-0.025	(-0.076, 0.026)	-0.023	(-0.065, 0.018)	**-2.505**	**(-4.743, -0.274)**	0.66	(-3.045, 4.359)	0.006	(-0.001, 0.014)
Birth weight z-score *	**0.216**	**(0.166, 0.266)**	**0.124**	**(0.09, 0.159)**	**0.136**	**(0.109, 0.165)**	**9.276**	**(7.766, 10.807)**	**4.64**	**(2.137, 7.171)**	0.003	(-0.003, 0.008)
Gestational age	0.003	(-0.021, 0.027)	0.005	(-0.012, 0.021)	-0.007	(-0.02, 0.006)	-0.001	(-0.727, 0.719)	-0.043	(-1.245, 1.151)	0	(-0.002, 0.003)
Maternal age	0	(-0.008, 0.007)	-0.004	(-0.009, 0.001)	-0.002	(-0.006, 0.002)	-0.153	(-0.373, 0.068)	-0.273	(-0.638, 0.092)	0	(-0.001, 0.001)
Pre-pregnancy BMI												
Overweight	-0.005	(-0.099, 0.088)	-0.034	(-0.098, 0.03)	0.039	(-0.013, 0.09)	0.579	(-2.232, 3.391)	1.545	(-3.111, 6.204)	-0.002	(-0.011, 0.007)
Obese	-0.03	(-0.131, 0.071)	-0.052	(-0.122, 0.017)	-0.009	(-0.065, 0.046)	-1.875	(-4.902, 1.138)	-1.782	(-6.794, 3.202)	0.001	(-0.009, 0.011)
Race/ethnicity												
Non-Hispanic White	-0.032	(-0.169, 0.105)	0.003	(-0.09, 0.097)	-0.029	(-0.105, 0.047)	-0.817	(-4.924, 3.297)	-1.362	(-8.155, 5.427)	-0.003	(-0.017, 0.011)
Non-Hispanic Black	-0.179	(-0.42, 0.063)	0.144	(-0.024, 0.315)	0.098	(-0.039, 0.236)	2.408	(-4.915, 9.77)	3.716	(-8.353, 15.851)	**-0.034**	**(-0.058, -0.01)**
Asian	0.005	(-0.176, 0.186)	-0.045	(-0.17, 0.08)	-0.051	(-0.152, 0.05)	-2.562	(-8.067, 2.943)	-4.546	(-13.697, 4.596)	-0.011	(-0.029, 0.008)
Other/Multiple race	-0.222	(-0.529, 0.085)	-0.071	(-0.292, 0.151)	**-0.183**	**(-0.363, -0.002)**	-4.745	(-14.88, 5.388)	-8.245	(-24.933, 8.452)	-0.023	(-0.055, 0.008)
Education												
Some college/Associate degree	0.003	(-0.109, 0.116)	-0.014	(-0.091, 0.063)	-0.026	(-0.088, 0.036)	-1.669	(-5.017, 1.682)	-3.729	(-9.259, 1.807)	-0.007	(-0.019, 0.004)
Bachelor's degree	0.06	(-0.081, 0.201)	-0.023	(-0.12, 0.074)	0.039	(-0.039, 0.118)	-0.004	(-4.238, 4.228)	0.665	(-6.325, 7.658)	0.004	(-0.01, 0.018)
Post-graduate degree	0.07	(-0.087, 0.227)	-0.02	(-0.128, 0.087)	0.067	(-0.02, 0.154)	1.127	(-3.607, 5.854)	2.223	(-5.603, 10.041)	0.001	(-0.014, 0.017)
Parity: Parous	0.073	(-0.017, 0.163)	**-0.069**	**(-0.131, -0.008)**	-0.03	(-0.08, 0.02)	-0.908	(-3.624, 1.803)	-2.068	(-6.554, 2.415)	0.005	(-0.004, 0.014)

Breastfeeding duration: more than 6 months	0.036	(-0.042, 0.113)	-0.023	(-0.077, 0.03)	**-0.052**	**(-0.095, -0.008)**	**-2.357**	**(-4.687, -0.024)**	**-5.117**	**(-8.976, -1.255)**	-0.001	(-0.008, 0.007)
Anthropometric z change *	**0.169**	**(0.139, 0.201)**	**0.127**	**(0.106, 0.15)**	**0.116**	**(0.098, 0.134)**	**8.329**	**(7.366, 9.325)**	**3.044**	**(1.449, 4.687)**	0.002	(-0.001, 0.005)

Notes: All models include time (age at visit in month) to adjust for child age; Bolded effect indicates statistically significance with a=0.05.

TKV is the total kidney volume for right and left kidney. Length, width, depth are average of left and right kidney measurement. Adjusted TKV is the ratio of tkv and body surface area. Adjusted kidney length is the ratio of kidney length and child height. Birth weight z-scores are sex and gestational week dependent and were calculated through INTERGROWTH-21st tool. Anthropometric z change is the difference of birthweight z-score and weight-for-age z-score.

Table S6: Adjusted LMMs for kidney outcomes (adjusted for maternal, fetal determinants, and postnatal growth) with log2-transformed cotinine level.

	Average Kidney Length (N=337)		Average Kidney Width (N=324)		Average kidney depth (N=325)		TKV (N=291)		Adjusted TKV (N=289)		Adjusted Kidney Length (N=308)	
Exposure	Estimate	95% CI	Estimate	95% CI	Estimate	95% CI	Estimate	95% CI	Estimate	95% CI	Estimate	95% CI
Intercept	**4.804**	**(3.204, 6.421)**	**2.744**	**(1.52, 3.979)**	**3.206**	**(2.363, 4.054)**	52.021	(-0.509, 106.275)	**124.216**	**(45.744, 205.513)**	**0.702**	**(0.557, 0.849)**
Child age at visit (month)	**0.033**	**(0.03, 0.035)**	**0.012**	**(0.01, 0.014)**	**0.016**	**(0.015, 0.018)**	**1.197**	**(1.102, 1.289)**	**0.36**	**(0.215, 0.499)**	**-0.002**	**(-0.002, -0.002)**
Child sex: female	-0.113	(-0.241, 0.014)	0	(-0.097, 0.097)	-0.023	(-0.091, 0.043)	-2.627	(-6.67, 1.348)	0.673	(-5.4, 6.647)	0.009	(-0.003, 0.02)
Birth weight z-score *	**0.208**	**(0.132, 0.285)**	**0.11**	**(0.053, 0.17)**	**0.14**	**(0.1, 0.18)**	**10.106**	**(7.757, 12.538)**	**4.501**	**(0.965, 8.153)**	0.001	(-0.006, 0.008)
Gestational age	0.012	(-0.027, 0.05)	0.013	(-0.017, 0.043)	-0.004	(-0.024, 0.017)	-0.01	(-1.337, 1.274)	0.478	(-1.509, 2.396)	0.002	(-0.001, 0.006)
Maternal age	0.007	(-0.005, 0.019)	-0.003	(-0.012, 0.006)	-0.001	(-0.008, 0.005)	-0.064	(-0.434, 0.309)	-0.051	(-0.605, 0.508)	0	(-0.001, 0.001)
Pre-pregnancy BMI												
Overweight	0.089	(-0.069, 0.246)	**-0.131**	**(-0.251, -0.011)**	-0.016	(-0.099, 0.066)	-1.206	(-6.131, 3.68)	-0.039	(-7.44, 7.304)	0.009	(-0.006, 0.023)
Obese	0.023	(-0.148, 0.193)	-0.129	(-0.257, 0.001)	-0.071	(-0.16, 0.017)	-4.023	(-9.201, 1.138)	-5.08	(-12.861, 2.631)	0.009	(-0.006, 0.024)
Race/ethnicity												
Non-Hispanic White	-0.005	(-0.242, 0.234)	0.01	(-0.172, 0.192)	-0.072	(-0.199, 0.053)	-1.381	(-8.795, 6.064)	-1.659	(-12.77, 9.398)	0.01	(-0.011, 0.031)
Non-Hispanic Black	-0.409	(-0.812, -0.008)	0.12	(-0.198, 0.447)	-0.047	(-0.271, 0.176)	-5.439	(-19.579, 8.705)	-10.535	(-31.549, 10.531)	**-0.07**	**(-0.107, -0.033)**
Asian	-0.045	(-0.339, 0.251)	0.04	(-0.189, 0.271)	-0.053	(-0.21, 0.103)	-2.091	(-11.171, 7.008)	-2.692	(-16.501, 11.069)	-0.019	(-0.045, 0.008)
Other/Multiple race	-0.013	(-0.414, 0.387)	-0.095	(-0.412, 0.223)	**-0.331**	**(-0.553, -0.11)**	-10.534	(-25.25, 3.985)	-17.91	(-39.807, 3.672)	-0.003	(-0.041, 0.035)
Education												
Some college/Associate degree	0.079	(-0.118, 0.275)	-0.016	(-0.163, 0.133)	-0.079	(-0.18, 0.023)	-1.98	(-7.925, 3.999)	-4.399	(-13.299, 4.54)	-0.008	(-0.026, 0.009)
Bachelor's degree	0.02	(-0.213, 0.254)	-0.08	(-0.259, 0.099)	0.014	(-0.111, 0.137)	-2.82	(-10.108, 4.431)	-4.025	(-14.9, 6.812)	-0.006	(-0.027, 0.014)
Post-graduate degree	0.052	(-0.203, 0.305)	-0.098	(-0.292, 0.097)	0.027	(-0.107, 0.162)	-1.646	(-9.695, 6.392)	-1.066	(-13.145, 10.98)	0.001	(-0.022, 0.024)
Parity: Parous	0.06	(-0.092, 0.212)	**-0.15**	**(-0.266, -0.032)**	-0.063	(-0.143, 0.018)	-3.672	(-8.507, 1.156)	-6.524	(-13.791, 0.722)	-0.005	(-0.018, 0.009)

Breastfeeding duration: more than 6 months	-0.037	(-0.172, 0.099)	-0.02	(-0.125, 0.082)	-0.026	(-0.097, 0.045)	-2.285	(-6.43, 1.895)	-5.341	(-11.567, 0.903)	-0.007	(-0.019, 0.005)
Anthropometric z change *	**0.141**	**(0.093, 0.189)**	**0.164**	**(0.127, 0.203)**	**0.13**	**(0.104, 0.158)**	**9.316**	**(7.743, 11.039)**	**3.556**	**(1.194, 6.116)**	0.002	(-0.002, 0.007)
Cotinine (log2)	**-0.024**	**(-0.045, -0.002)**	0.011	(-0.005, 0.027)	0	(-0.011, 0.011)	-0.059	(-0.724, 0.613)	-0.031	(-1.026, 0.975)	-0.001	(-0.003, 0)

Notes: All models include time (age at visit in month) to adjust for child age; Bolded effect indicates statistically significance with a=0.05.

TKV is the total kidney volume for right and left kidney. Length, width, depth are average of left and right kidney measurement. Adjusted TKV is the ratio of tkv and body surface area. Adjusted kidney length is the ratio of kidney length and child height. Birth weight z-scores are sex and gestational week dependent and were calculated through INTERGROWTH-21st tool. Anthropometric z change is the difference of birthweight z-score and weight-for-age z-score.

Table S7: Baseline characteristics for the analytic sample and the CHES sample.

	Analytic sample (N=892)	Not included (N=2041)	P-Value
**Maternal baseline characteristics**			
Maternal age at recruitment: Mean (SD)	31.77 (5.65)	32.34 (5.40)	0.003
Pre-pregnancy BMI	26.42 (23.03, 30.52)	25.02 (22.00, 29.15)	< 0.001
Normalweight	348 (40.2%)	998 (50.0%)	
Overweight	277 (32.0%)	570 (28.6%)	
Obese	241 (27.8%)	428 (21.4%)	
Maternal race/ethnicity			< 0.001
Hispanic	582 (65.9%)	814 (40.7%)	
Non-Hispanic White	188 (21.3%)	801 (40.0%)	
Non-Hispanic Black	32 (3.6%)	115 (5.7%)	
Asian	64 (7.2%)	208 (10.4%)	
Other/Multiple race	17 (1.9%)	64 (3.2%)	
Marital status			0.438
Married/living with a partner	781 (87.7%)	1751 (88.7%)	
Divorced/separated/single/widowed	110 (12.3%)	224 (11.3%)	
Education			< 0.001
High school or less	413 (47.1%)	476 (25.2%)	
Some college but no degree/Associate degree	138 (15.7%)	267 (14.1%)	
Bachelor's degree	160 (18.2%)	469 (24.8%)	
Post-graduate degree	166 (18.9%)	678 (35.9%)	
Empolyed	483 (54.1%)	1363 (69.4%)	< 0.001
Insurance type			< 0.001
Public	568 (65.0%)	839 (41.5%)	
Private	306 (35.0%)	1183 (58.5%)	
Parity			< 0.001
Nulliparous	332 (37.3%)	992 (48.7%)	
Parous	558 (62.7%)	1043 (51.3%)	
GDM	237 (26.6%)	334 (17.2%)	< 0.001
HDP	149 (16.7%)	250 (12.9%)	0.006

Chronic hypertension	36 (4.0%)	84 (4.1%)	0.925
Preeclampsia	85 (9.6%)	123 (6.4%)	0.002
Eclampsia	0 (0.0%)	1 (0.1%)	0.498
PIH	49 (5.5%)	85 (4.4%)	0.194
Smoking during pregnancy	0 (0.0%)	26 (1.4%)	< 0.001
Urine cotinine (ng/mL)	0.02 (0.01, 0.08)	0.02 (0.01, 0.14)	0.069
Alcohol use during pregnancy	146 (16.4%)	299 (15.7%)	0.621
Hospital recruitment site			< 0.001
Bellevue	97 (10.9%)	206 (10.1%)	
NYU Brooklyn	493 (55.3%)	593 (29.1%)	
NYU Manhattan	290 (32.5%)	1229 (60.2%)	
Other	12 (1.3%)	13 (0.6%)	
**Child baseline characteristics**			
Gestational age at delivery	39.29 (38.25, 40.04)	39.14 (38.29, 40.00)	0.471
Birth weight	3313.50 (2990.00, 3600.00)	3260.00 (2920.00, 3570.00)	0.013
Birth weight Z-Score: Mean (SD)	0.27 (0.96)	0.18 (0.98)	0.013
PTB	66 (7.4%)	176 (8.6%)	0.265
LBW	62 (7.0%)	157 (8.0%)	0.388
SGA	46 (5.2%)	136 (6.9%)	0.088
Child sex			0.412
Male	462 (51.8%)	1018 (50.1%)	
Female	430 (48.2%)	1012 (49.9%)	
Breastfeeding more than 6 mo			< 0.001
Less than six months	335 (38.8%)	681 (49.2%)	
More than six months	529 (61.2%)	704 (50.8%)	

Notes: For maternal age and birth weight Z-score,mean (SD) is provided. For other continuous variables, median (IQR) is provided. For categorical variables, number (percentage) is provided. Birth weight z-scores are sex and gestational week dependent and were calculated through INTERGROWTH-21st tool.

Missing data, n(%): Not included (N=2041): Pre-pregnancy BMI (kg/m^2), 45 (2.20%); Prenatal BMI categories, 45 (2.20%); Maternal race/ethnicity, 39 (1.91%); Marital status, 66 (3.23%); Education, 151 (7.40%); Employment, 76 (3.72%); Insurance type, 19 (0.93%); Parity, 6 (0.29%); GDM, 104 (5.09%); HDP, 98 (4.80%); Chronic hypertension, 2 (0.10%); Preeclampsia, 104 (5.09%); PIH, 104 (5.09%); Smoking during pregnancy, 132 (6.47%); Urine cotinine (ng/mL), 1262 (61.82%); Alcohol use during pregnancy, 133 (6.51%); Birth weight (g), 73

(3.58%); Birth weight Z-Score, 78 (3.82%); LBW, 73 (3.58%); SGA, 78 (3.82%); Child sex, 11 (0.54%); Breastfeeding more than 6 months, 656 (32.12%).

Missing data, n(%): Analytic sample (N=892): Pre-pregnancy BMI (kg/m^2), 26 (2.91%); Prenatal BMI categories, 26 (2.91%); Maternal race/ethnicity, 9 (1.01%); Marital status, 1 (0.11%); Education, 15 (1.68%); Insurance type, 18 (2.02%); Parity, 2 (0.22%); GDM, 2 (0.22%); HDP, 2 (0.22%); Chronic hypertension, 2 (0.22%); Preeclampsia, 2 (0.22%); PIH, 2 (0.22%); Urine cotinine (ng/mL), 518 (58.07%); Alcohol use during pregnancy, 2 (0.22%); Birth weight (g), 12 (1.35%); Birth weight Z-Score, 13 (1.46%); LBW, 12 (1.35%); SGA, 13 (1.46%); Breastfeeding more than 6 months, 28 (3.14%).

Table S8: Intraclass Correlation Coefficients of renal measurements measured by two readers in the quality control process.

**Renal measurement**	**N**	**ICC**
Right renal length	95	1
Left renal length	87	1
Right anterior-posterior	93	0.99
Left anterior-posterior	84	0.99
Right transverse	93	0.96
Left transverse	84	0.99

Figure S1. Boxplot of continuous covariates considered.

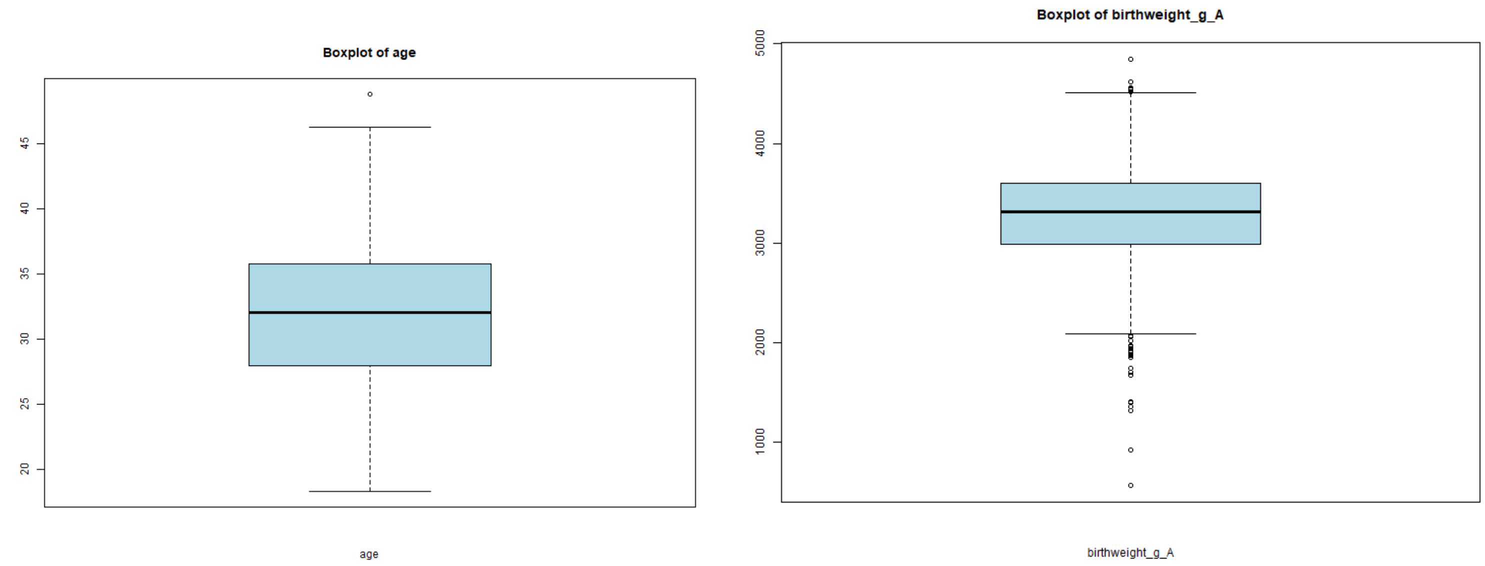

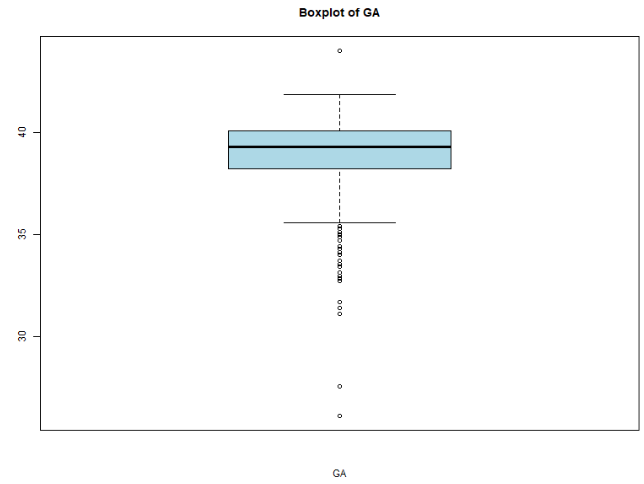
Boxplot of GA
30
35
40
GA

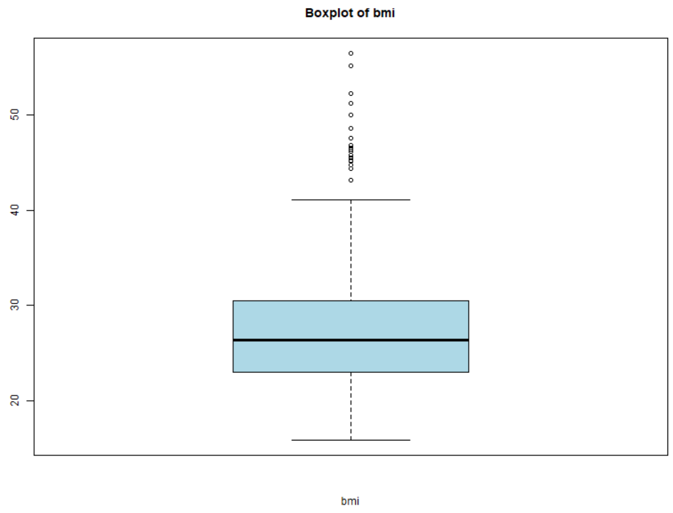
Boxplot of bmi
20
30
40
50
bmi

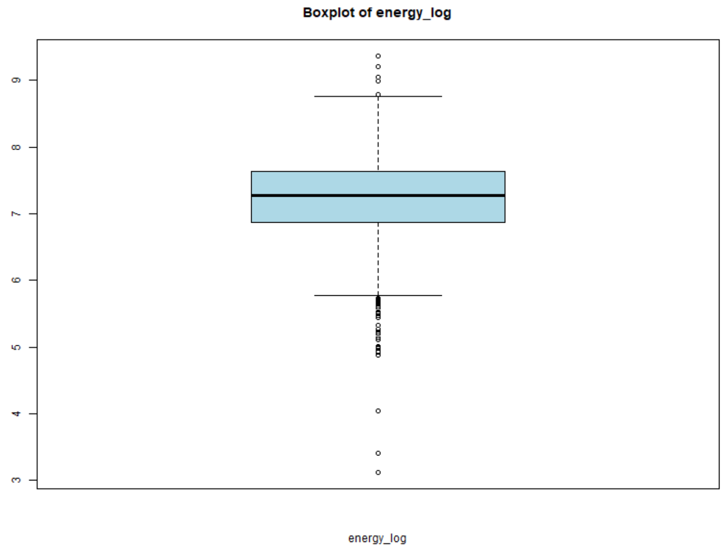
Boxplot of energy_log
3
4
5
6
7
8
9
energy_log

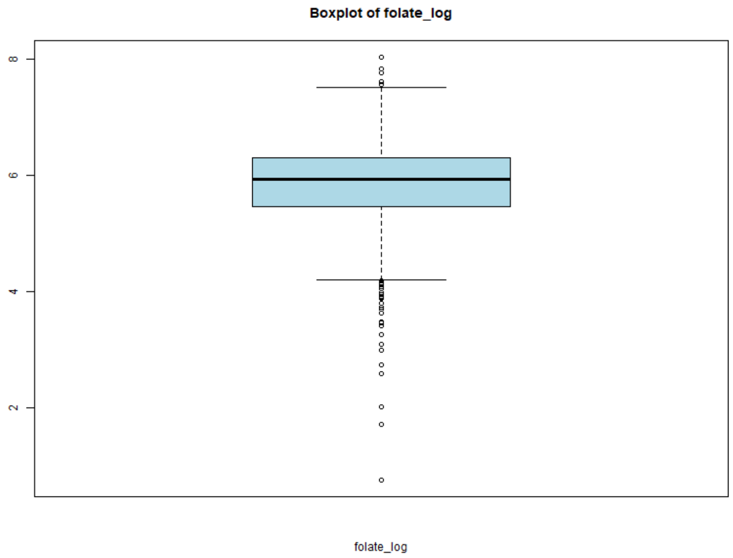
Boxplot of folate_log
2
4
6
8
folate_log

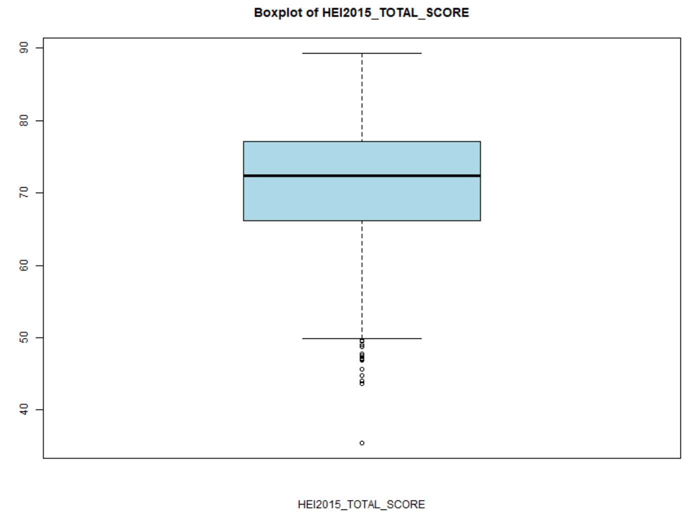
Boxplot of HEI2015_TOTAL_SCORE
90
80
70
60
50
40
HEI2015_TOTAL_SCORE

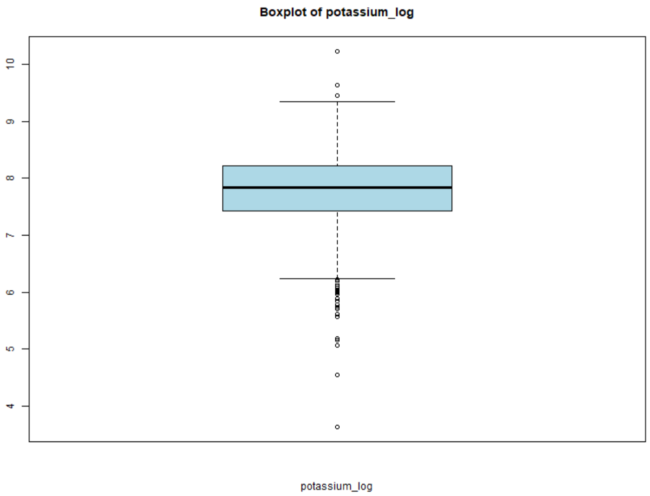
Boxplot of potassium_log
10
9
8
7
6
5
4
potassium_log

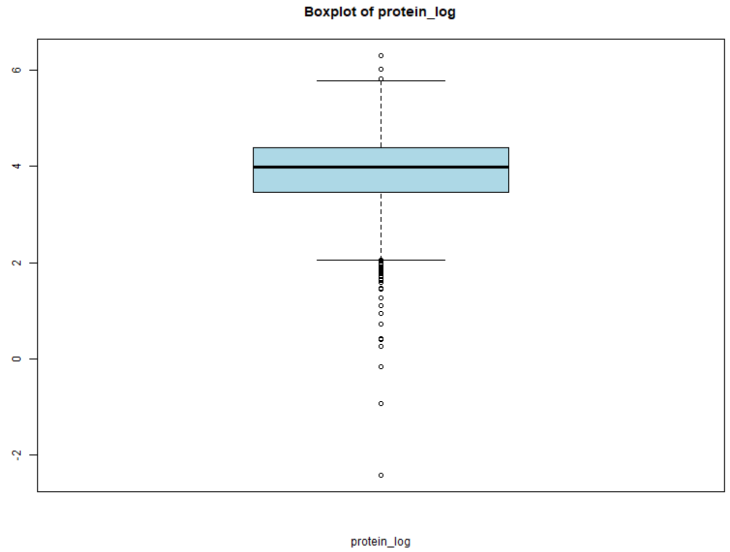
Boxplot of protein_log
6
4
2
0
-2
protein_log

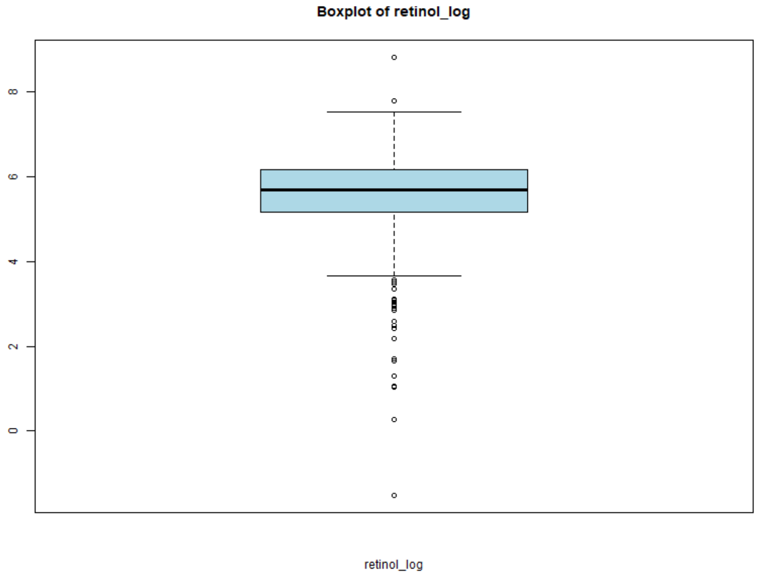
Boxplot of retinol_log
8
6
4
2
0
retinol_log

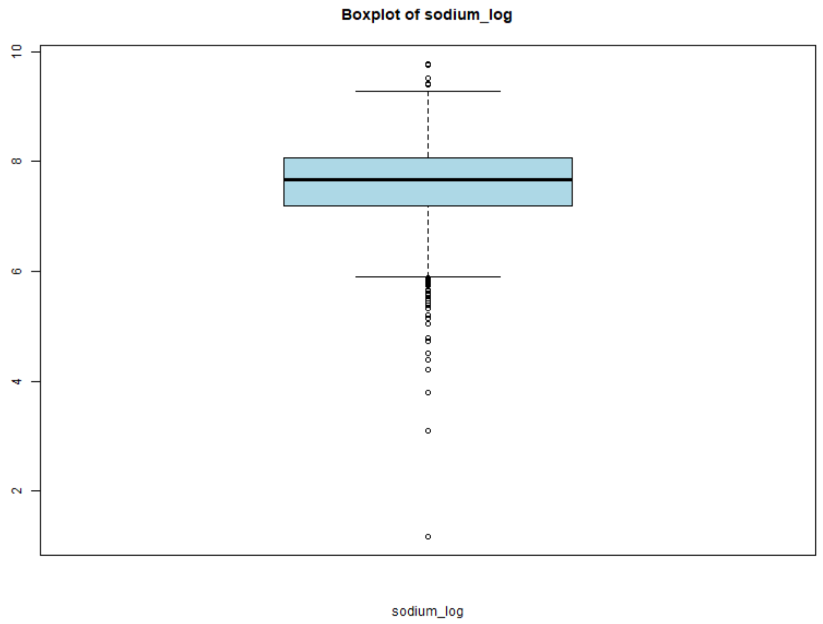

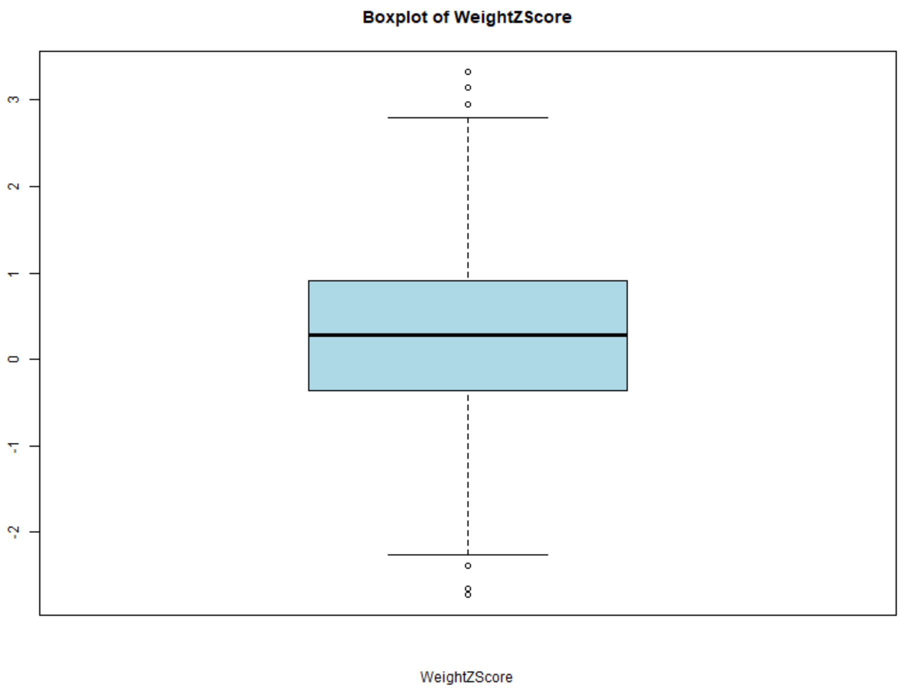

Figure S2. Boxplot of outcome measures by timepoint.

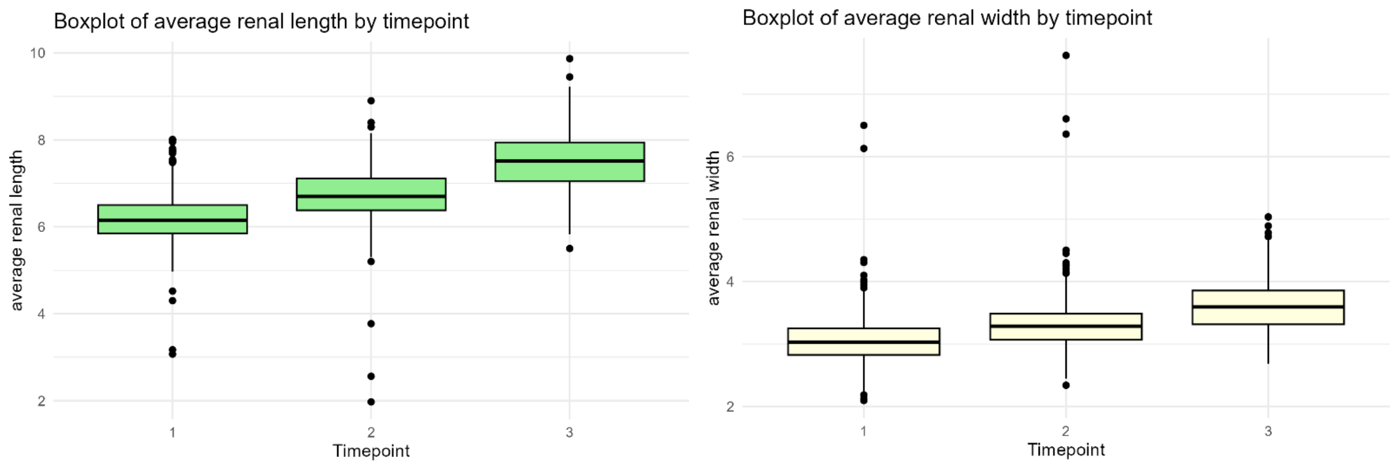

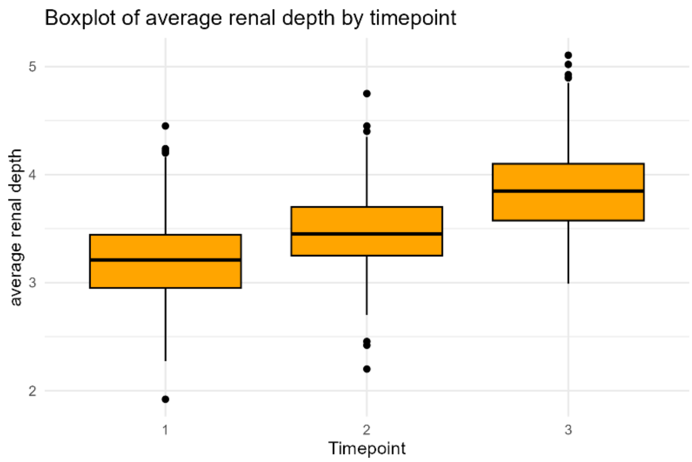
Boxplot of average renal depth by timepoint
average renal depth
5
4
3
2
1
2
3
Timepoint

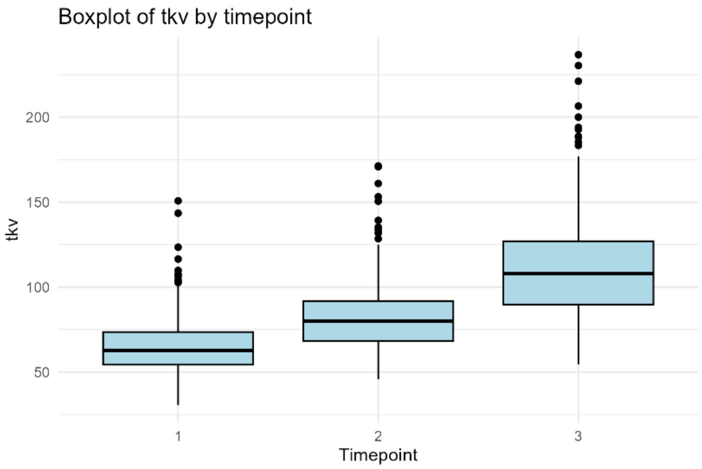
Boxplot of tkv by timepoint
tkv
200
150
100
50
1
2
3
Timepoint

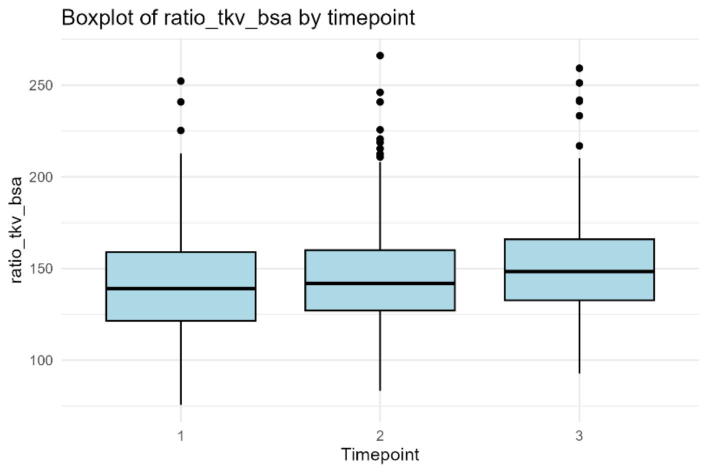
Boxplot of ratio_tkv_bsa by timepoint
ratio_tkv_bsa
250
200
150
100
1
2
3
Timepoint

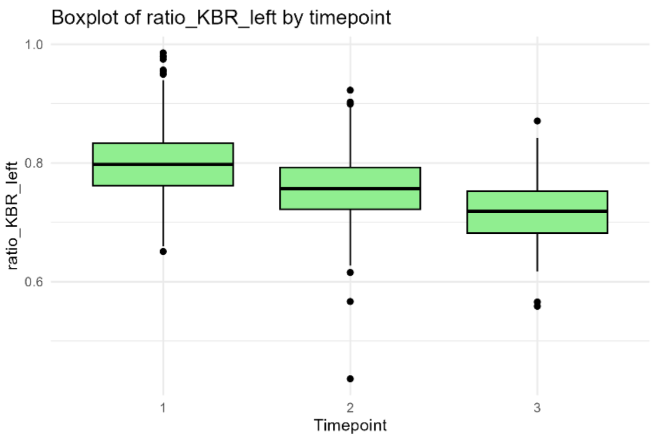
Boxplot of ratio_KBR_left by timepoint
ratio_KBR_left
1.0
0.8
0.6
1
2
3
Timepoint

Figure S3. Correlation coefficient matrix for all continuous variables.

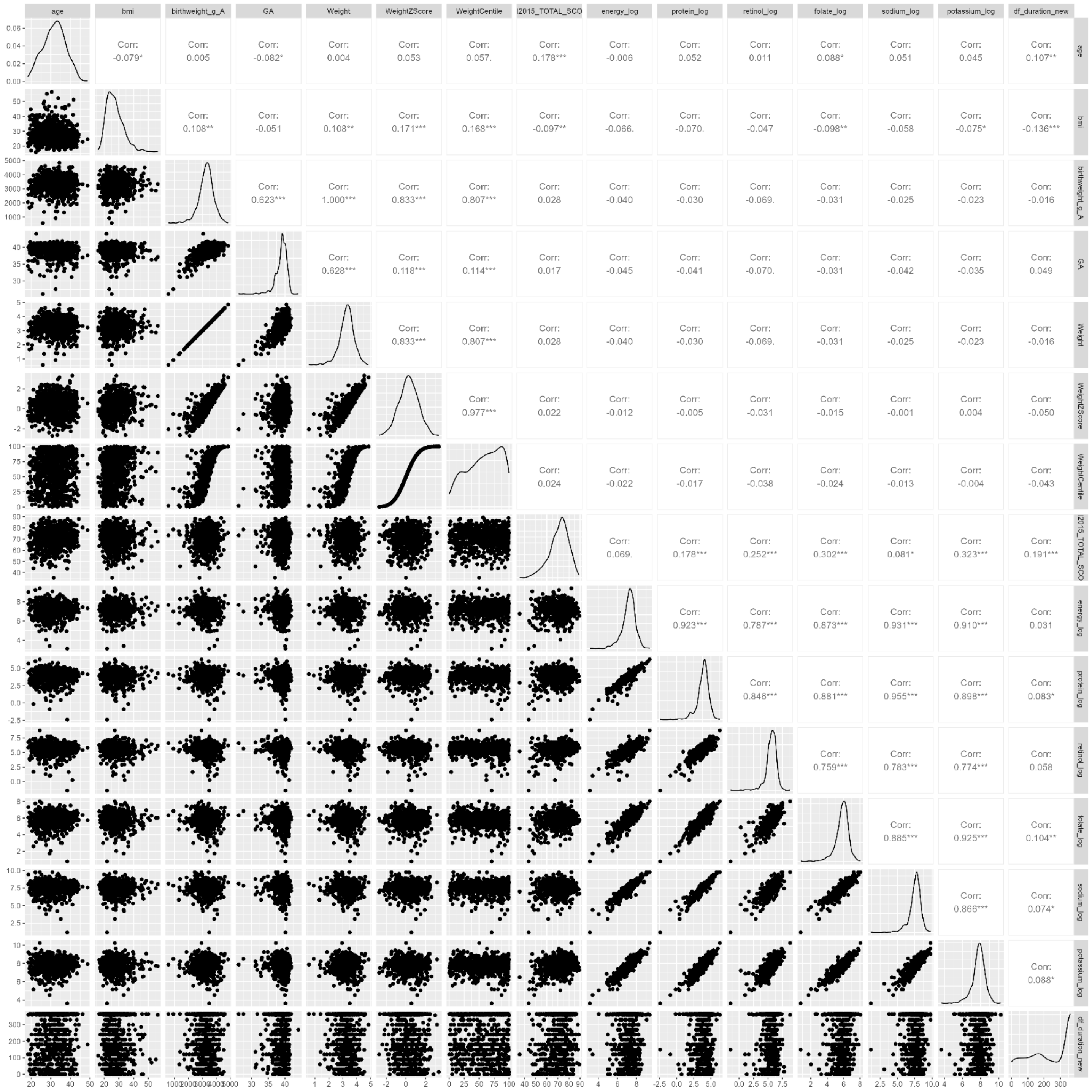

age
bmi
birthweight_g_A
GA
Weight
WeightZScore
WeightCentile
I2015_TOTAL_SCO
energy_log
protein_log
retinol_log
folate_log
sodium_log
potassium_log
df_duration_new
